# The acceptability of photovoice as a method for incorporating resilience-enhancing factors into pediatric pain research

**DOI:** 10.1371/journal.pone.0272504

**Published:** 2022-09-02

**Authors:** Elizabeth Donovan, Katherina Tanson, Sarah R. Martin, Georgia Weston, Anya Griffin, Lonnie K. Zeltzer

**Affiliations:** 1 Department of Psychology, Simmons University, Boston, Massachusetts, United States of America; 2 David Geffen School of Medicine at University of California Los Angeles, Los Angeles, California, United States of America; 3 Charles R. Drew University of Medicine and Science, Los Angeles, California, United States of America; 4 Department of Anesthesiology & Perioperative Care, University of California Irvine School of Medicine, Orange, California, United States of America; 5 Creative Healing for Youth in Pain, Los Angeles, California, United States of America; 6 Stanford University School of Medicine, Palo Alto, California, United States of America; 7 Departments of Pediatrics, Anesthesiology, Psychiatry and Biobehavioral Sciences, David Geffen School of Medicine at University of California Los Angeles, Los Angeles, California, United States of America; University of Illinois at Urbana-Champaign, UNITED STATES

## Abstract

Recurrent or chronic pain affects 11–38% of children and adolescents. Pediatric pain research typically focuses on risk factors, such as anxiety and parent functional disability, but resilience-building, protective factors also play an important role in the pain experience. New methods to incorporate resilience-enhancing factors into pain research are needed. Photovoice is a highly participatory research method, where participants take photos to address a common question, caption their photos, and discuss the meaning of the photos in a group. The main objective of this study was to determine whether photovoice is an acceptable method to young people living with chronic pain for identifying and sharing sources of joy. Another objective was to explore sources of joy. Sixteen adolescents and young adults participated, which involved meeting in a group to discuss the goal of the study, taking photographs of self-identified sources of joy over a two-week period, and meeting as a group again to discuss the photographs and participate in a focus group about the experience. Results suggest that photovoice is an acceptable method, as all participants took photographs and attended both meetings, and three themes from the focus group data suggested the participants considered photovoice to be appropriate: 1.) Relief associated with meeting peers, 2.) Potential to benefit young people living with pain, and 3.) Potential to raise awareness. Three themes emerged from the discussion of the photographs to describe sources of joy: 1.) Gratitude for everyday pleasures and accomplishments, 2.) Support from pets, and 3.) Journey of acceptance. Results add to the strengths-based literature on pediatric pain by identifying an acceptable method that could be further explored for use as an intervention to enhance protective factors such as positive affect, gratitude, and social support and to compare the experiences of different populations of youth living with pain.

## Introduction

Pediatric pain is common, with prevalence rates suggesting 11–38% of children and adolescents experience recurrent or chronic pain, and approximately 5% experience significant pain-related dysfunction [1]. In recent years, pediatric pain research has tended to focus on critical risk factors such as patient pain intensity, anxiety, pain coping, and parent functional disability as associated with the pain experience [2], with less research focusing on protective, resilience-building factors (e.g., acceptance and optimism). However, these factors also play an important role in the pain experience and can be fostered in young people to optimize living with chronic pain [3].

The ecological resilience-risk model in pediatric chronic pain focuses on individual and familial/social strengths [3]. Individual factors such as trait mindfulness, trait positive affect, and optimism may be protective for young people living with pain. For example, optimism predicted better quality of life via minimizing pain-related fear and catastrophizing in a sample of youth with chronic pain [4]. When confronting pain, factors such as pain acceptance and pain-related self-efficacy may also enhance resilience. In a study among youth with chronic headache, pain self-efficacy in tandem with pain acceptance was associated with less disability, better school functioning, and fewer depressive symptoms, and predicted less functional disability [5]. Youth living with chronic pain also benefit from positive social environments, such as families where active coping is modeled, and social support from peers [6]. Finding methods to promote resilience-enhancing factors among youth living with pain could provide opportunities to support this population [3].

Photovoice is a highly participatory research method which may be well-suited to exploring resilience-enhancing factors among youth living with pain. With roots in the action research tradition [7], the method involves participants taking photos to address a common question, captioning their own photos, and discussing the meaning of the photos in a group. The original goals of photovoice were to allow people to record their community’s strengths and concerns, reflect on issues through discussion, and foster change by reaching policymakers [8]. In the last fifteen years, it has been increasingly used in healthcare research [9, 10]. For example, HIV-vulnerable and low-income adolescents in Botswana used photovoice to raise awareness of risks and engage in education and prevention efforts [11]. Only one study has used photovoice with youth living with chronic pain. In this study, a sample of youth living with sickle cell disease (SCD), reflected on their experiences with SCD using photovoice, including the impact of SCD, coping with the disease, and the importance of family and support [12].

Photovoice may be an ideal method for incorporating resilience-enhancing factors into pediatric pain research. First, the act of participating in a photovoice study can be empowering [10]. Second, while photovoice has typically been used to reveal needs or concerns among people living with a health condition, the approach can be adapted to specifically focus attention on factors that may enhance resilience such as optimism, savoring/mindfulness, or positive affect. Third, the process of sharing and discussing photographs has the potential to promote social support [10].

Learning whether the photovoice approach is appropriate is important as it could be used in future pediatric pain, strengths-based research, for example, to structure an intervention focused on protective factors, and learning more about sources of joy among youth with pain will also add to the strengths-based literature on pediatric pain. As such, the main objective of this study was to determine whether young people living with chronic pain would find the photovoice method to be an acceptable approach for identifying and sharing sources of joy. The focus on joy was chosen as savoring positive experiences to enhance positive affect may be protective for young people living with pain and has been identified as a dimension within the ecological resilience-risk model in need of further exploration [3]. The second objective was to explore sources of joy among youth living with chronic pain.

## Methods

### Design

This study used photovoice, a method guided by a participatory action research framework [13] where participants take photos to address a common question, caption their own photos, and discuss the meaning of the photos in a group [8]. The study followed the procedure for conducting photovoice studies outlined by Wang and Burris [8]. The ecological resilience-risk model in pediatric chronic pain which emphasizes the importance of individual and familial/social strengths [3] informed our research question. We chose this model because of our focus on protective factors and our interest in determining the acceptability of a method to explore resilience-enhancing strengths such as those outlined in the model (e.g., optimism, positive affect, mindfulness, and social support).

We used Sekhon et al.’s [14] definition of acceptability: “A multi-faceted construct that reflects the extent to which people delivering or receiving a healthcare intervention consider it to be appropriate, based on anticipated or experienced cognitive and emotional responses to the intervention.” (1 p. 4). Based on Sekhon et al.’s recommendation, acceptability was measured with behavior and self-report. Drawing on other photovoice studies, we used 1.) attendance and photo-taking [15, 16] and 2.) qualitative data from focus groups [17]. We adhered to guidelines for conducting [18, 19] and reporting [20] qualitative research.

The research team included academic researchers and clinicians. The team had expertise in qualitative research (ED), previous experience with Photovoice (AG), expertise in working clinically with youth with chronic pain (SM, GW, AG, LKZ) and conducting pediatric pain research (ED, SM, GW, AG, LKZ). Author KT was a third-year medical student.

### Participants

Sixteen young people living with chronic pain ranging in age from 14 to 24 (eight were 14 to 17 years of age and eight were 19 to 24 years of age), participated in the study. (Please see Table 1). We chose this age range as a better understanding of biological growth, in combination with delayed role transitions, such as completing education, marriage, and parenthood, have expanded the conceptualization of the time period between childhood and adulthood [21]. Likewise, pediatric care may continue into the 20s [22]. As such, inclusion criteria were to 1.) be between 13 and 25 years of age, 2.) have a self-reported, physician diagnosis of chronic pain, and 3.) speak fluent English. The exclusion criterion was a refusal to give informed consent.

**Table 1:** Participant characteristics.

	Adolescents	Young adults	Total
	n = 8	n = 8	n = 16
Age	15.5 (1.20)	22.13 (1.55)	18.81 (3.67)
Age range	14–17	19–24	
Sex			
Male	1	1	2
Female	7	7	14
Ethnicity			
Hispanic/Latino	1	0	1
Non-Hispanic/Non-Latino	7	8	15
Race			
White	7	6	13
Black/African-American	0	0	0
Asian	0	2	2
Multi-Racial	0	0	0
Unknown	1	0	1

Note: Data are presented as Mean (SD) for continuous variables and N for categorical variables

All 16 participants self-identified as someone who has chronic pain. Participants had the option to disclose a specific diagnosis or to remain under the generalized context of chronic pain. Eleven out of the 16 participants chose to reveal additional details about their health/chronic pain, which included: migraines, headaches, irritable bowel syndrome, complex regional pain syndrome, cancer pain, neuropathic pain, conversion disorder, and fibromyalgia.

### Recruitment

Participants were recruited using convenience sampling in three ways. Author LKZ reached out to current and former patients of an outpatient chronic pain clinic. The same author also emailed the study flier to other pediatric pain physicians in her network. The opportunity to participate in the study was also advertised through social media channels associated with the non-profit organization founded by author LKZ.

When a young adult or caregiver of an adolescent sent an email of interest, a research team member (Author ED) responded with inclusion criteria questions. If a potential participant (or caregiver of a potential participant under 18 years of age) screened into the study, Author ED responded with a consent form (for the young adult or caregiver), followed by an assent form when a caregiver’s written consent was received. Of the sixteen people who contacted the research team to express interest in participating, all sixteen young people were enrolled into the study and placed into one of two groups based on age (< or >18 years of age). Participants were then sent an email with information about how to join the first virtual meeting.

### Procedure

The study involved two online meetings. Participants took photos in the period between the meetings (Table 2). In keeping with guidelines for focus group size [23] and recognizing the difference in experiences of young people who would typically be living with parents versus those typically living independently, the meetings with each of the two age-based cohorts were conducted separately. The first virtual meeting lasted 90 minutes and was conducted using Zoom for Healthcare, which is HIPAA compliant. As well as eight participants (and guardians of participants in the group consisting of participants under 18 years), three female research team members with training in qualitative research methods, and no prior relationship with the participants, attended the meetings. Two of the research team members served as group co-facilitators (Author GW and Author ED), and one team member took notes (Author KT).

**Table 2:** Study phases.

Phase	Description
1: Instructions	Participants given research question to guide photo-taking, instructions, and ethical guidelines.
2: Taking photos	Over a two-week period, participants take photos that address the research question and select two photos to share with the group.
3: Discussion of photos and photovoice experience	Participants each present and discuss their photos using the SHOWeD method, participate in a group discussion about themes in photos, and participate in a focus group about the photovoice process and how it could be used in the future.

The main goal of the first meeting was to prepare participants for the photo-taking period. At the beginning of the meeting, each member of the group and the three team members introduced themselves by talking about something they loved to do. The team members described their backgrounds but did not disclose any further information about personal reasons for conducting the study. A co-facilitator (Author ED) then gave an overview of the photovoice procedure and introduced the research question, “What brings me joy?”, informed by the ecological resilience-risk model in pediatric chronic pain (3). Participants were instructed to answer the research question by taking daily photos over a two-week period. One of the co-facilitators (Author GW) also presented guidelines for taking photographs adapted from previous studies (e.g., photos should be de-identified) [24, 25]. Please see Table 3. Participants were instructed to choose two photographs at the end of the two weeks, and to email the photographs to one of the co-facilitators. Immediately after the meeting, participants were sent an email that contained the following: 1.) A $30 Amazon e-gift card, 2.) instructions and ethical guidelines for taking photos, 3.) a summary of the questions that a co-facilitator would ask them about their two photos during the second meeting, and 4.) a request to prepare a caption for each of the two photos to be shared with the group.

**Table 3:** Ethical guidelines give to participants, adapted from [24].

Do not take a picture that identifies someone.
Get permission. If you want to include a person in your photo, you must get verbal consent from that person (or from a group of up to four people). Explain that the photos will not identify them.
No trespassing. Pictures should be taken on public property. Ask permission to take pictures on private property
Do not take pictures of illegal activities
Do not to take photographs that stigmatize, embarrass, or shame individuals or groups
Don’t hide. You or the camera should not be hidden while taking pictures. Explain what you are doing if anyone asks why you are taking pictures
Respect privacy. Do not take pictures that invade another’s privacy.

The two-hour, second meetings, with the same people in attendance, were held two weeks later. During the first part of the meeting, one of the co-facilitators (Author GW) displayed the participants’ photos and asked each participant in turn about their two photographs. As discussed in Wallerstein & Bernstein [26] and modified by Valenzuela et al. [12] for pediatric pain research, the co-facilitator used the SHOWeD approach, asking: What do you SEE here? What’s really HAPPENING? (to you, to your feelings) How does the story relate to OUR lives? (How do you feel about it?) WHY does this strength exist? How can we become EMPOWERED by this? What does this say about your chronic pain? After each participant had responded to the questions, they were asked to offer captions for each of their photographs, which were added to the slide deck of photos. After each individual answered questions about their own photographs, the participants discussed the categories of ideas common to the photographs as a group.

During the last 30 minutes of the meeting, participants were asked to participate in a focus group led by Author ED, the goals of which were to understand acceptability of the photovoice approach, including the experience of taking photos, sharing the photos with peers, and beliefs about how the approach could be used to benefit young people with chronic pain. Author ED created an interview guide to address these goals; two members of the research team reviewed the interview guide, and any feedback was incorporated into a final draft. (Please see Table 4). Video recordings of the focus groups were later transcribed.

**Table 4:** Interview guide to explore acceptability of photovoice method among youth living with chronic pain.

Questions	Prompts
Why did you decide to take part in the Photovoice project?	What was your motivation for taking part?
	Did you participate in other advocacy activities in your community before the photovoice project (e.g., advisory board. . .)?
Thinking back about the Photovoice project, what experience did you have?	What were the activities or moments that you remember the most (and why)?
	What did you learn from the other participants?
Thinking about the aim of the project, how would you describe to someone else what you learned?	Did it change the way you think about healing?
	Are there things, that you now know, but before weren’t aware of?
	To do with your thoughts or beliefs?
	Your relationships?
	Your community?
	Media? Society?
	Were there topics that came up, that you did not have in mind?
If you learned something in the photovoice project, can you give an example on how you apply/use this knowledge now?	Is there anything you might do differently?
	Do you think projects like photovoice can create further initiatives among the people who participate? Example?
	Have you had any ideas for your own projects (individually or with others)?
	Do you feel like there is something that does not allow you to apply and maintain what you have taken from the experience?
Did you know the other participants before, or did you get to know them there?	Are you still keeping in touch?
	When you went out to take the photos, did you talk to the people you took photos of?
	Did they ask you what you were doing?
Do you think this process could help to build community among young people living with pain?	
Let’s talk about your participation in the project… How did you feel? What did you learn about yourself in the project? What, if anything, changed about you throughout the project?	Describe the experience of watching other people view your photos.
	Did you do things that surprised you about yourself?

After the meeting, a $50 Amazon e-gift card for participating in the second group meeting was emailed to participants (>18 years) and to parents of participants who were under 18 years. The categories of responses identified by the participants were also emailed to the participants to confirm that their thoughts had been accurately captured. No participant disagreed with the categories. The entire research team then met to discuss the focus groups and agreed thematic saturation had been reached and that no further data were required. The study was conducted in August 2020 and was approved by the Institutional Review Board at Simmons University in Boston, MA.

### Photo exhibit

In keeping with Wang and Burris’s original conceptualization of photovoice as a way to share information with the wider community [8], photographs and captions were exhibited at a showcase event hosted by the nonprofit founded by Author LKZ which is dedicated to supporting youth living with pain and their families.

### Measures

The two behavioral measures of acceptability were attendance at the two meetings and submitting photographs. Drawing on other photovoice studies [15, 16], the acceptability threshold was over 70% of participants taking photos and over 70% of participants attending both photovoice sessions.

The qualitative, self-report measure of acceptability was whether themes from the focus groups indicated acceptability of the photovoice approach using our definition of acceptability [14] (i.e., is photovoice appropriate for documenting and discussing sources of joy, based on anticipated or experienced cognitive and emotional responses?)

### Data analysis

The goals of the data analysis were to 1.) determine acceptability of the photovoice approach among young people living with chronic pain, and 2.) explore factors that promote joy. The behavioral data (attending both meetings and submitting two photographs) were analyzed by calculating frequencies. The broad approach to analyzing the qualitative data was essentialist/realist, which reports experiences, meanings, and the reality of participants [18]. We chose this inductive technique because we were interested in learning more about daily experiences of the participants and believed that imposing a framework a priori would not be helpful in meeting this goal. To organize the data, we used a templated organizing style [27].

The transcribed focus groups were the basis for the analysis. Specifically, the focus group about experience in the photovoice study informed our description of acceptability of the photovoice approach. The participants’ discussion of the photos and captions informed our description of sources of joy. Our interest in the photographs centered around how the images were interpreted by the participants. As others have described [28], poly textual thematic analysis for visual data was used as inspiration for how to approach an analysis that includes images [29]. In this context, the photos were used to illustrate what the participants described in words about sources of joy, and the analysts referred to the photos while reviewing the transcript.

In keeping with the philosophy of the photovoice approach [8], participants are sometimes involved in data analysis [9]. We incorporated participants to the degree that was feasible for this study. During the second meeting, participants were asked to generate and come to consensus on main categories that emerged from the discussion of the photographs and captions. Participants were also invited to comment via email on the accuracy of the categories after the meeting [19]. These categories were then incorporated into the data analysis associated with sources of joy.

The specific data analysis steps, informed by Braun and Clarke [18] including features of image-analysis [30], were as follows. First, as noted in the procedure, after listening to each participant caption and discuss their two photos, participants agreed on main categories that were common to many members of the group. Second, two of the co-facilitators independently read the transcripts several times, referring to photos and field notes, and created a coding structure, adapting the participant-generated categories to serve as main codes. Three additional codes were added to address acceptability of the photovoice method (Please see Table 5). Third, two members of the research team (ED and KT) independently applied the codes to the transcripts. During two, hour-long discussions, the two analysts discussed application of the codes and came to final consensus on the codes. Fourth, the two analysts, and the other co-facilitator of the groups (Author GW), met with two pediatric pain clinicians (Authors SM and AG) to discuss themes emerging from data associated with each of the codes. At the end of the meeting the five research team members came to consensus on three overarching themes to describe the participants’ experience of joy, as well as three themes to describe acceptability of the photovoice methodology, and whether these themes indicated acceptability. Fifth, two analysts (Authors ED and SM) selected vivid excerpts from the transcripts to illustrate main themes.

**Table 5:** Codes used to analyze transcripts.

Research question	Codes
Acceptability	Motivation for participating
	Experience participating
	Future benefits
Sources of joy	Gratitude
	Social support
	Self-care
	Self-image

## Results

### Acceptability

#### Attendance and photo taking

All 16 participants attended both meetings and submitted two photographs.

#### Main themes associated with acceptability of the photovoice method

To determine acceptability of the photovoice method, we identified three main themes emerging from the focus groups centering around the experience of participating in the photovoice project and beliefs about how the method could be used in the future: 1.) Relief associated with meeting peers, 2.) Potential to benefit young people living with pain, and 3.) Potential to raise awareness.

*Relief associated with meeting peers*. Most participants described that their motivation for participating in the study was to meet and share pain management strategies with peers. Some adolescents expressed that they didn’t know other young people who had chronic pain but felt that they could benefit from meeting peers who could understand their experience. Participants reported overwhelmingly that they found the experience of meeting peers to be valuable.

*It’s great being able to talk to someone who gets it even a little bit more than anybody else and I think that you bond over that shared experience*. *So*, *I think this is a great place to do that too*. Young Adult, 04

Adolescents, in particular, expressed gratitude for the experience of meeting other young people living with pain, for example:

*When you’re in pain and then others around you aren’t like that*, *you feel alone*. *But*, *when you go to something like this you see many other kids around your age dealing with some sort of pain like yours*, *not necessarily the exact*, *but still in a lot of pain*. *It makes you feel kind of comforted… not really*, *but it gives you more like other people are going through this and it encourages you… okay*, *I guess to be like stronger*, *if that makes sense*. Adolescent,16

*Potential to benefit young people living with pain*. Adolescents and young adults expressed that the approach could be used to help individuals cope, as one young adult described:

*It’s not only about making other people understand*, *but also understanding yourself because you’re not always fully aware of what’s going on and with creative expression you kind of stumble across things where you didn’t know that this might’ve been a trigger or this is a strategy that helps you*, *so it’s building more and more awareness of your feelings over time*. Young Adult, 01

Another young adult explained that the approach had the potential to cause a shift in perspective through documenting and reflecting on their experiences:

*I think for me the thing I’ve gotten most out of this was the fact that it allowed me to be reflective in a different way than I haven’t ever been before and to realize those things that bring me joy*, *whereas doing a puzzle I never really thought of it like that before*. *Like I knew it was a distraction*, *but I never really thought of it as something that brought me joy and there’s just so many things that after two weeks*, *after reflecting on the word joy*, *I guess makes me realize how many more things that I have in my life that I can be thankful for*. Young Adult, 06

Young adults saw an opportunity to use the approach to support adolescents as they learned to manage pain, as one person explained:

*I’m hoping through this project we can encourage people in high school and maybe even middle school have to kind of I guess a call to action so to speak*. *Like hear their voices or thoughts*. *Everyone has a phone*, *generally*. *But taking a picture is much easier than having to write an essay or a short novel about what you’ve been going through*. Young Adult, 02

Another young adult explained that the approach could be used to mentor adolescents:

*I think these pictures can help making it easier to become mentors to younger people with chronic pain that we once were*. Young Adult, 05

*Potential to raise awareness*. Participants also discussed ways that this approach could be used to raise awareness of the benefits of a holistic approach to pain management, as one adult described:

*From a policy level*, *I can imagine taking a more holistic approach to health care in general*. *Pharmacologically*, *medications help manage your symptoms and your pain*, *but it doesn’t fix like your mentality towards pain*. *So*, *having different mediums*, *like creative arts or art therapy could really help coping mentally with your condition*. Young Adult, 07

### Exploration of sources of joy

#### Main themes

**Fig 1:**
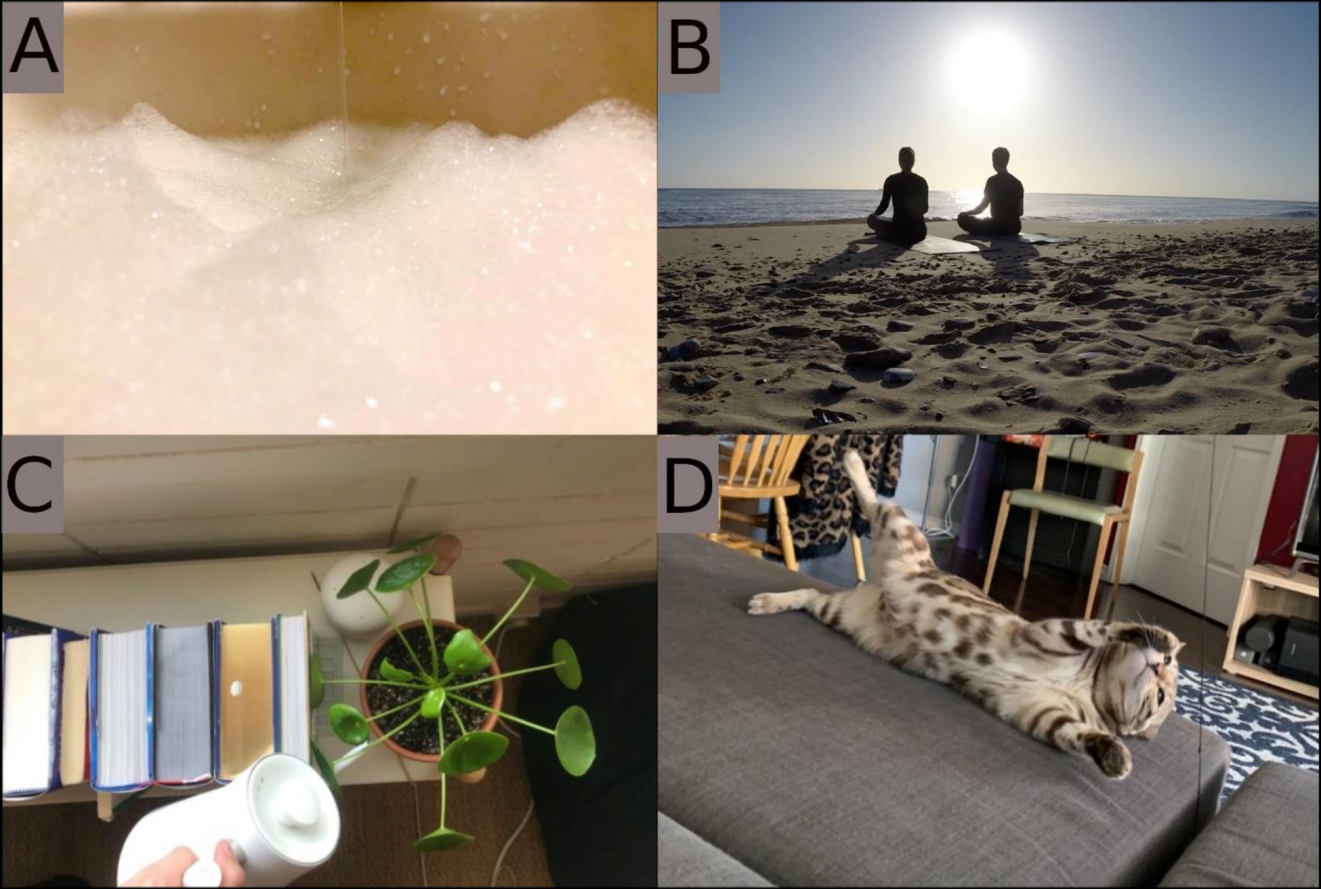
Participant photos. A. “When the world turns red and responsibilities pile ahead, look to the bubbling hub; surprise, it’s your own tub” B. “Morning routine” C. “Taking care of other life” D. “You can always make me laugh”.

**Fig 2:**
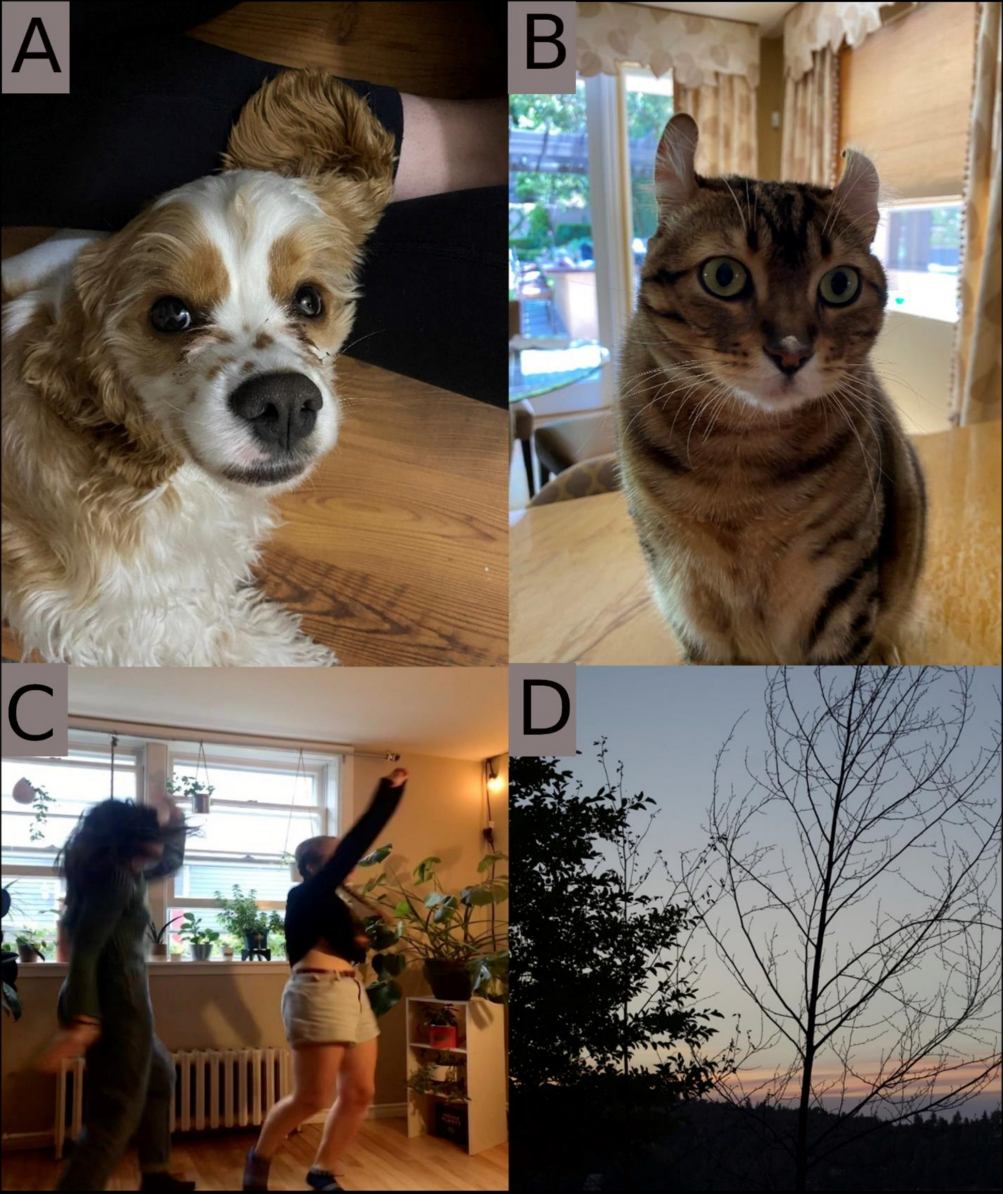
Participant photos. A. “Unconditional love” B. “Flour power” C. “Two peas in a wonky little pod” D. “Sunset Road”.

Three main themes emerged from the discussions of captions and photographs of sources of joy: 1.) Gratitude for everyday pleasures and accomplishments, 2.) Support from pets, and 3.) Journey of acceptance. These same themes emerged in both the adolescent and young adult groups, although there were some developmental differences, highlighted below. Please see Figs 1 and 2 for example photos and captions. Photos and captions provided by participants as well brief descriptions of all thirty-two photos are included as [S2 File].

*Gratitude for everyday pleasures and accomplishments*. Most participants expressed pleasure derived from everyday activities, such as baths (appreciation for this simple pleasure is expressed in a caption in Fig 1A) and cooking, often commenting that their experience with chronic pain had given them a unique perspective on how these activities could not be taken for granted. Taking the time to savor these small, joyful pleasures lead to feelings of gratitude, as one young adult explained:

*Small things are things you can be grateful for and you might have a different perspective on it because of your pain than other people*. *For them it might just be normal*, *or they might be unaware that it could be something* positive. Young Adult, 01

As well as the pleasure of experiencing these moments, some participants also expressed joy from everyday accomplishments that others might take for granted:

*This was the first time I put dishes away in a while*, *just coincidently*. *I really like to organize*. *It’s really pleasing*. *And I was just so grateful to myself and all the progress that I’ve made*. Adolescent, 10

*Support from pets*. All participants described joy resulting from their relationships. Social support came from a variety of sources, including friends and family, although it was overwhelmingly described as coming from relationships with pets. Pets brought joy for many reasons. Some participants commented that their pets’ playfulness brought joy to their lives, as one young adult noted (Fig 1D):

*It’s not necessarily my cat sitting there makes me happy*, *but when she’s playful or being goofy that just heightens the level of happiness that I have*. Young Adult, 06

And

*[He] does dumb*, *playful stuff all the time* (Fig 2B), *and now that we have [another pet] as well who has not let me alone this entire time*. *It’s just a reminder that we can still experience you know playfulness even though it’s not necessarily through ourselves*. Young Adult, 04

Another participant described the positive role of the family pet:

*We’ve had [our dog] since I was young and when I first initially got chronic pain*. *My parents got her because our family dynamic was so sad so that we needed someone to cheer us up… as I got the dog I realized you can’t play with the dog if you can’t walk [laughs]*. *Or you can but crutches are complicated*. *And so I started walking because of her*. Young Adult, 05

Others noted a certain ease in their relationship with their pets that was not always present in their relationships with humans. This acceptance brought joy, as one adolescent noted:

*[My dog] can make me feel better in a way that a human necessarily can’t*. *Like a human can say the wrong thing or do the wrong thing and a dog can’t really and will have unconditional love for you and will always be there for you no matter what*. Adolescent, 14

An adolescent described receiving comfort (Fig 2A) during times of distress:

*When it gets really bad I can just be a wreck essentially and like someone or something will be there and comfort me even if they can’t necessarily do everything they want to*. Adolescent, 16

While pets were discussed most frequently, support from family and friends also brought joy. Support from friends who also experienced pain was described by one young adult as a cherished understanding:

*Um*, *I feel really happy looking at it now*. *It was a really happy moment for both of us* (Fig 2C). *We both deal with chronic pain*. *Like she has chronic pain in most of her joints and we really connected over that and been able to support each other through it*. *And having a little dance it out session is what we do when we’re feeling down or sad about it*. Young Adult, 08

*Journey of acceptance*. Participants described joy through their journey of acceptance. There were developmental differences, with younger participants discussing the process of learning how to care for oneself and older participants discussing a shift in self-image. One adolescent participant described a growing sense of confidence in her ability to manage her pain:

*For me it’s kind of like looking back at how empty I felt* (Fig 2D). *Like looking at everyone having the time of their lives and the tree really is an example of me in that way*. *But now I’m a tree bearing more leaves as I grow*. *The sun is setting on each day*, *I grow a little more*. Adolescent, 11

An adolescent described joy from learning to care for herself:

*I think once you accept that it is part of your life now you can sort of kind of start to see what you can do to help yourself*. *I feel like I have a pretty set list of things I can do to help myself in the moment but it’s definitely a journey to get those things*. *It takes a lot of time to figure out what works*, *a lot of time*. Adolescent, 09

One young adult described a growing sense of self-awareness and a shifting self-image:

*I think it says that even if chronic pain does define my life in a lot of ways*, *it doesn’t define everything about who I am*. *Because it’s so unpredictable living with pain*. *There are still things that I can have as a constant in my life*, *like my plants* (Fig 1C). *And like moving out on my own was an exciting thing*. *There are still things you can do even if your life is unpredictable that can make you feel stable*. Young Adult, 08

## Discussion

Participants in our study found the photovoice approach to be acceptable, as they took photographs and attended both meetings, describing benefits resulting from the experience and suggesting future applications of the methodology. The participants also shared a range of sources of joy. Given that resilience-enhancing factors, such as savoring and positive affect, play an important role in the pain experience [3], findings from this study suggest that photovoice holds promise as a method for incorporating these types of factors into pain research with teens and young adults.

All the adolescents and young adults in this study attended the first virtual meeting, took photographs of joyful moments, and shared two of their photographs with the group two weeks later. Themes from the focus groups around acceptability of the photovoice methodology suggested that participants were motivated to participate as a way to meet peers and to share, and learn about, pain management strategies. Many young people living with chronic pain experience isolation and loneliness, in part due to feeling different than their same age peers, and not understood by same-aged peers who do not experience pain [31]. The participants in our study expressed that they experienced the social support they were seeking; this was especially true for the younger, adolescent, participants who expressed relief and happiness from meeting other young people who could relate to their experience of living with pain. Overall, findings from the focus group suggest that it may be acceptable to use the photovoice approach in the way described in this study.

Themes emerging from the acceptability focus groups and the discussion of photos and captions also suggest other ways the photovoice method could be used to support young people living with pain. In research with other populations, photovoice has been used to structure social support groups [32], and the results from our study suggest that the photovoice method could be further explored as the basis of a peer-based, social support group for young people living with pain. The participants in our study described appreciation for the opportunity to connect with peers, and a willingness to reflect on their journey of learning to live with pain. Pain is difficult to put into words and using photography as a form of self-expression may be a useful tool for communicating with peers about a complicated and challenging experience. Young adult participants also commented that the method could be used to mentor younger people.

The results of our study suggest that photovoice could also be used to structure an intervention, particularly around gratitude. In the focus groups centering on acceptability, some participants described experiencing a shift in perspective as a result of participating in the study. Looking for moments of joy was described as increasing gratitude for everyday pleasures. Indeed, the discussion of sources of joy centered around gratitude for taking a bath, cooking, or helping in the kitchen, events which could be challenging at times, but were not taken for granted. Recent studies have reported the benefits of positive-psychology-informed approaches to enhancing wellbeing in adult chronic pain patients [33, 34]. One intervention included components focused on self-compassion, savoring joyful moments, and gratitude [33]. Photovoice may provide an acceptable approach to young people with pain for structuring an intervention focused on resilience-enhancing factors such as these.

We aimed to mitigate limitations associated with qualitative research by following standard qualitative evaluative criteria [19]. To strengthen the internal validity of the findings, researchers with different backgrounds analyzed the data. We attempted to minimize bias by enlisting researchers familiar with the study population, but not involved with analysis, to review the methodological approach and participate in data analysis. The inclusion of participants of different ages allowed us to understand different perspectives. In addition, participants were included in the data analysis to the extent that was possible: generating initial categories and confirming them as accurate.

An important next step is to continue to document and compare the range of diverse experiences of young people living with pain. The participants in our study were mostly female, white, and cisgender. We did not collect information about socioeconomic status or how long participants had lived with chronic pain. The importance of race, ethnicity, and socioeconomic status in research on child health is well-documented [35]. Photovoice is ideal for revealing unique, lived experiences, particularly with a phenomenon such as pain which relies on self-report but is often challenging for young people to describe with words [36]. In future research, posting recruitment information in a wider range of community and healthcare settings may help to increase the diversity of potential participants. In addition, being open to conducting group meetings online ensures that getting to a different location is not a barrier, and others [37] have recommended budgeting for cameras to ensure that owning a phone or camera is not a barrier to participation.

In summary, photovoice has the potential to be a creative, engaging, and acceptable way to incorporate resilience-building factors [3, 4] into research with young people living with pain by offering opportunities for social support and a method for enhancing protective factors such as gratitude and savoring of positive moments as part of an overall approach to supporting young people living with pain.

## Supporting information

S1 File Limited data set. (XLSX)

S2 File Photos, and captions shared by participants and descriptions of photos. (DOCX)

S3 File Consent and assent forms. (PDF)
