# Supplementary material for: The acceptability of photovoice as a method for incorporating resilience-enhancing factors into pediatric pain research
Source: PLoS One. 2022 Sep 2;17(9):e0272504. doi: 10.1371/journal.pone.0272504 (PMC9439202; doi:10.1371/journal.pone.0272504)
Supplement: S2 File — (DOCX) [file pone.0272504.s002.docx]

**Photos and captions shared by participants with descriptions of photos**

| **Photo** | **Description** | **Caption** |
| --- | --- | --- |
| **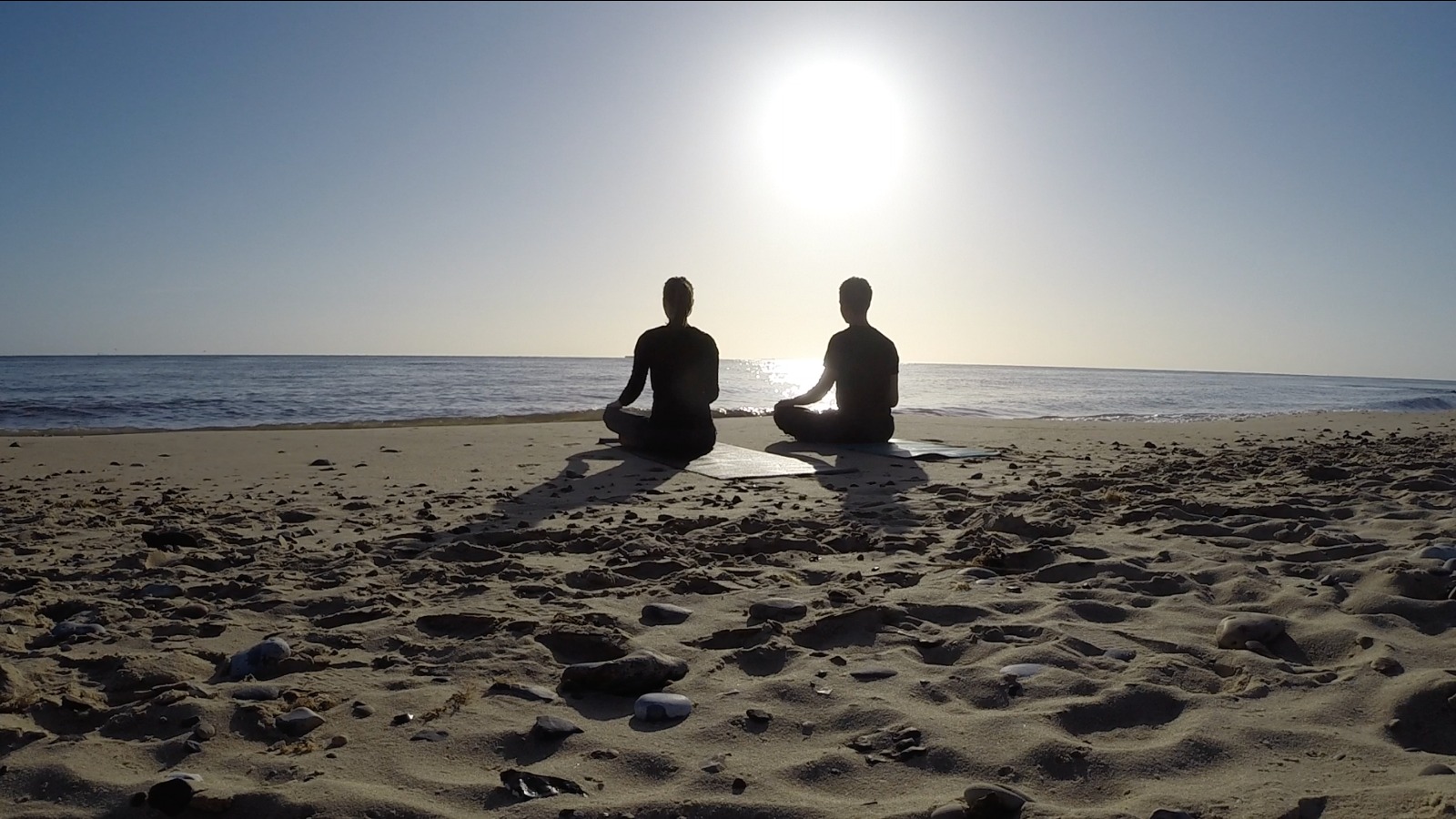** | Two individuals on the beach engaging in yoga while the sun illuminates their silhouettes. | *“Morning Routine”* |
| **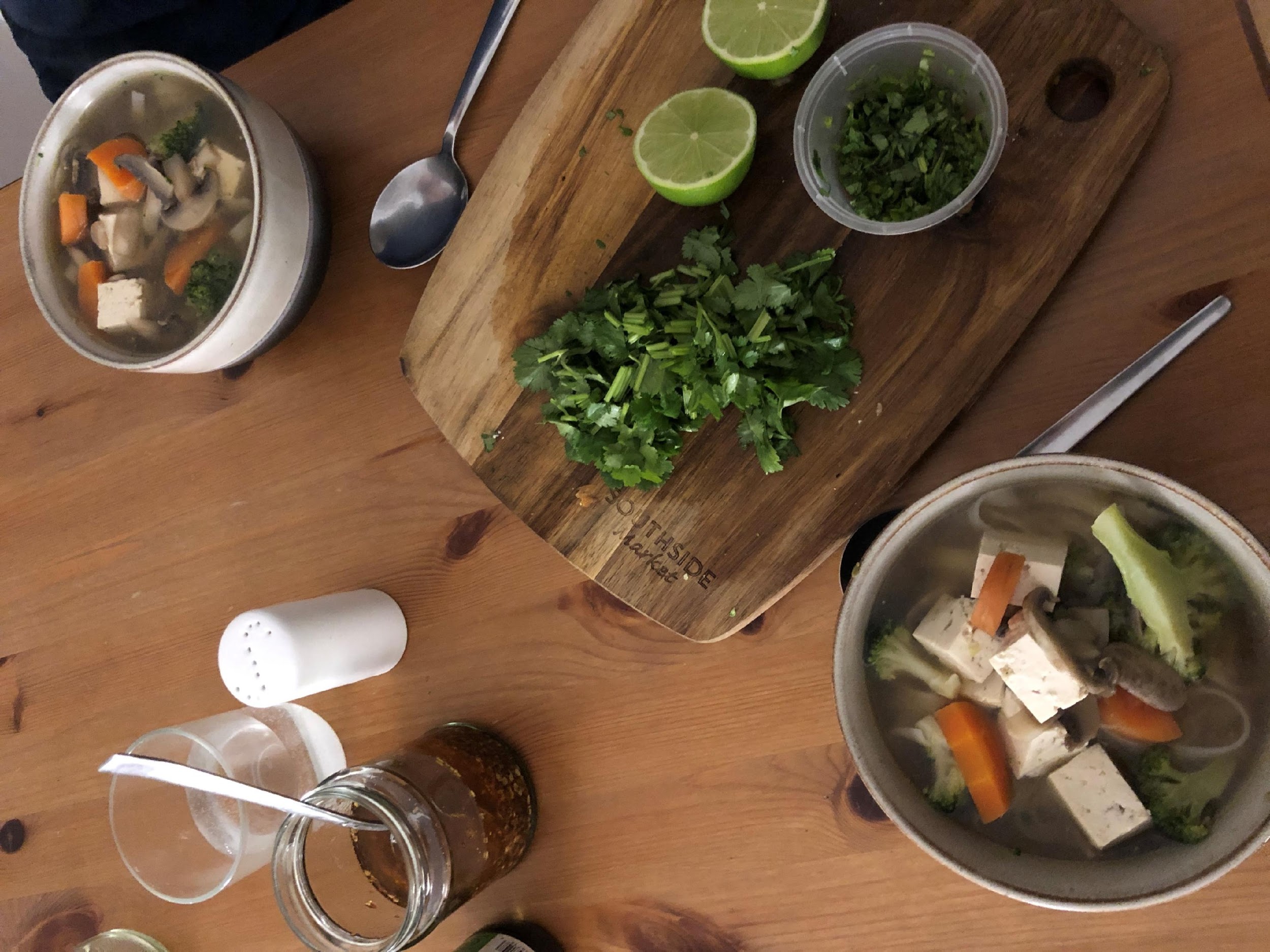** | Preparation of a meal featuring various vegetables and soup. | *“Mindless Cooking.”* |
| **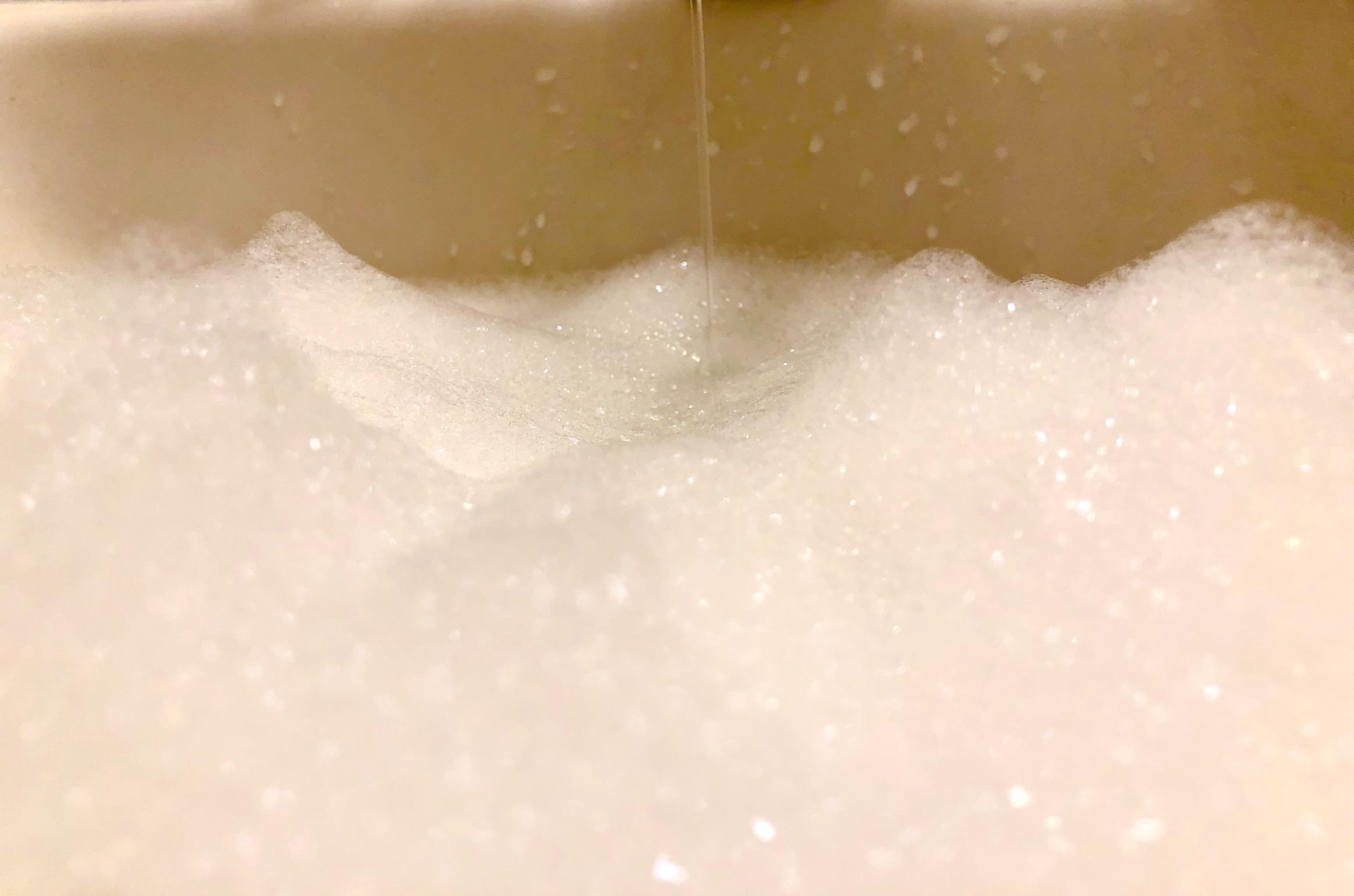** | Overflowing bubbles within a tub. | *“When the world turns red and responsibilities pile ahead, look to the bubbling hub; surprise, it’s your own tub.”* |
| **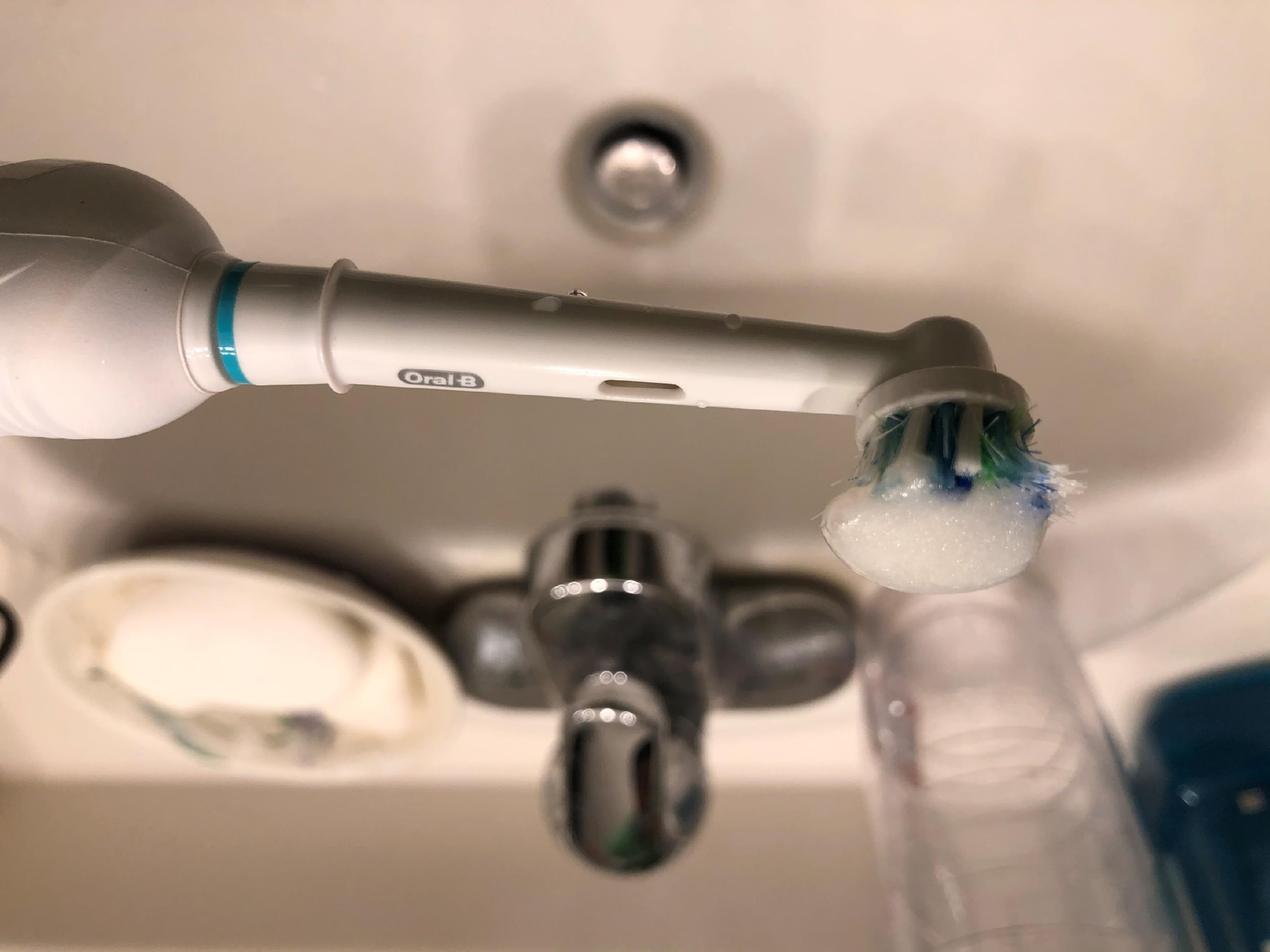** | Electric toothbrush with white tooth paste held in front of sink that has a glass and soap bar on its edge. | *“Many take it for granted. They see pedestrian paste and a routine brush, we see purposeful haste and a loving touch.”* |
| Photo removed as participant may be identifiable | A smiling individual with someone’s arm around them as they stand in the pool. | *“Defying Gravity.”* |
| **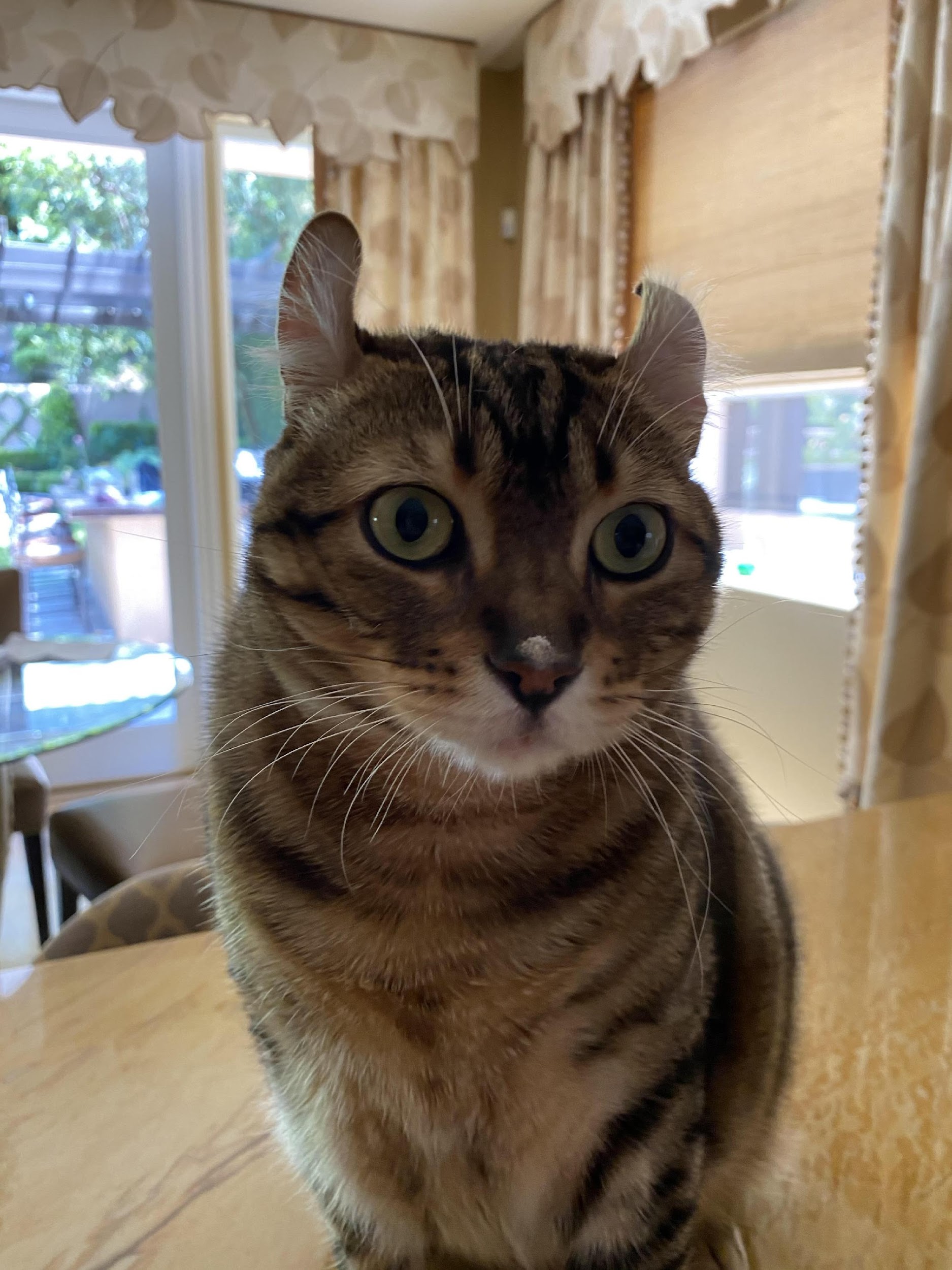** | Staring kitten with flour on its nose. | *“Flour Power.”* |
| **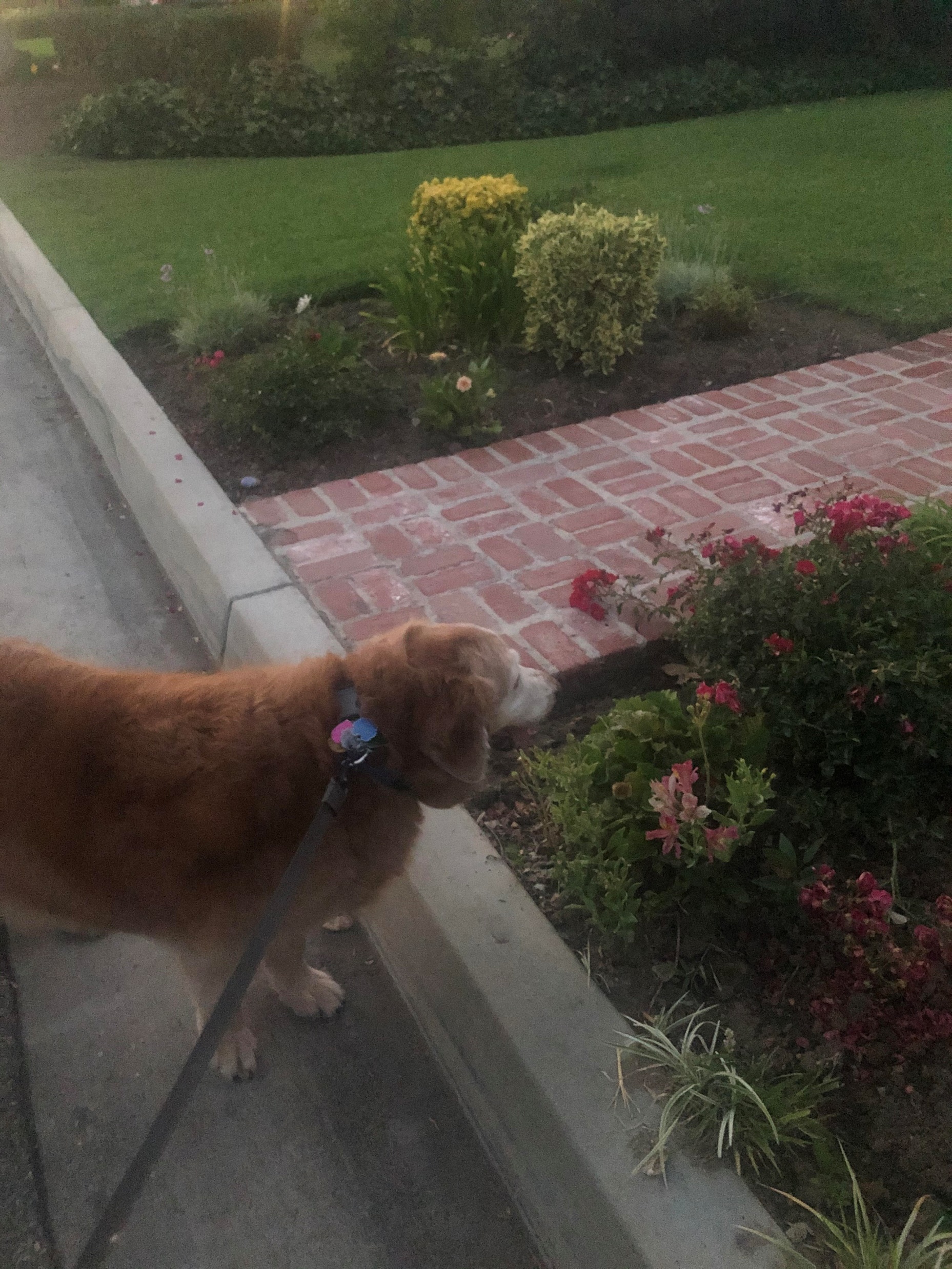** | A dog being walked on a leash stopped in front of a rose bed. | *“Chronic pain isn’t stopping us from smelling the roses.”* |
| **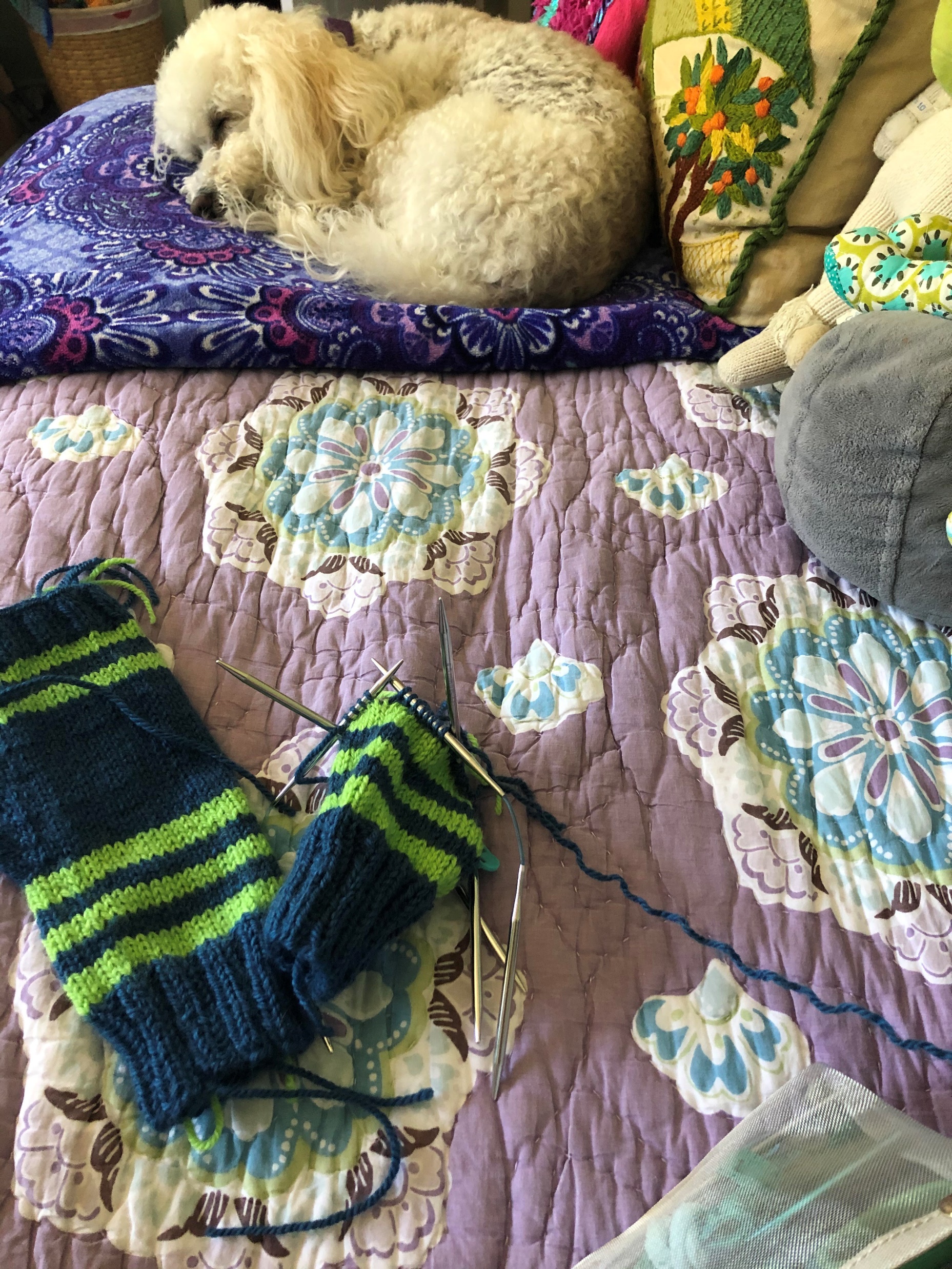** | Blue and green stripped knitwear on a floral purple blanket, with a dog lying in the background. | *“Cozy knitting sudoku, plus one.”* |
| **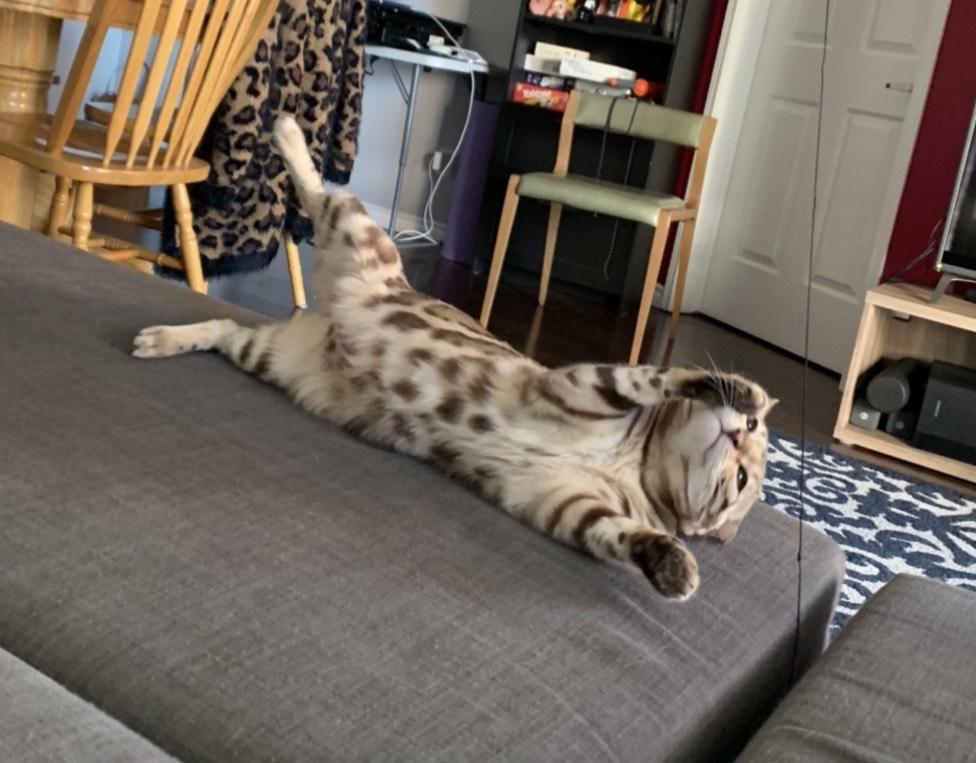** | Kitten lying on a gray chair stretching its entire body. | *“You can always make me laugh.”* |
| **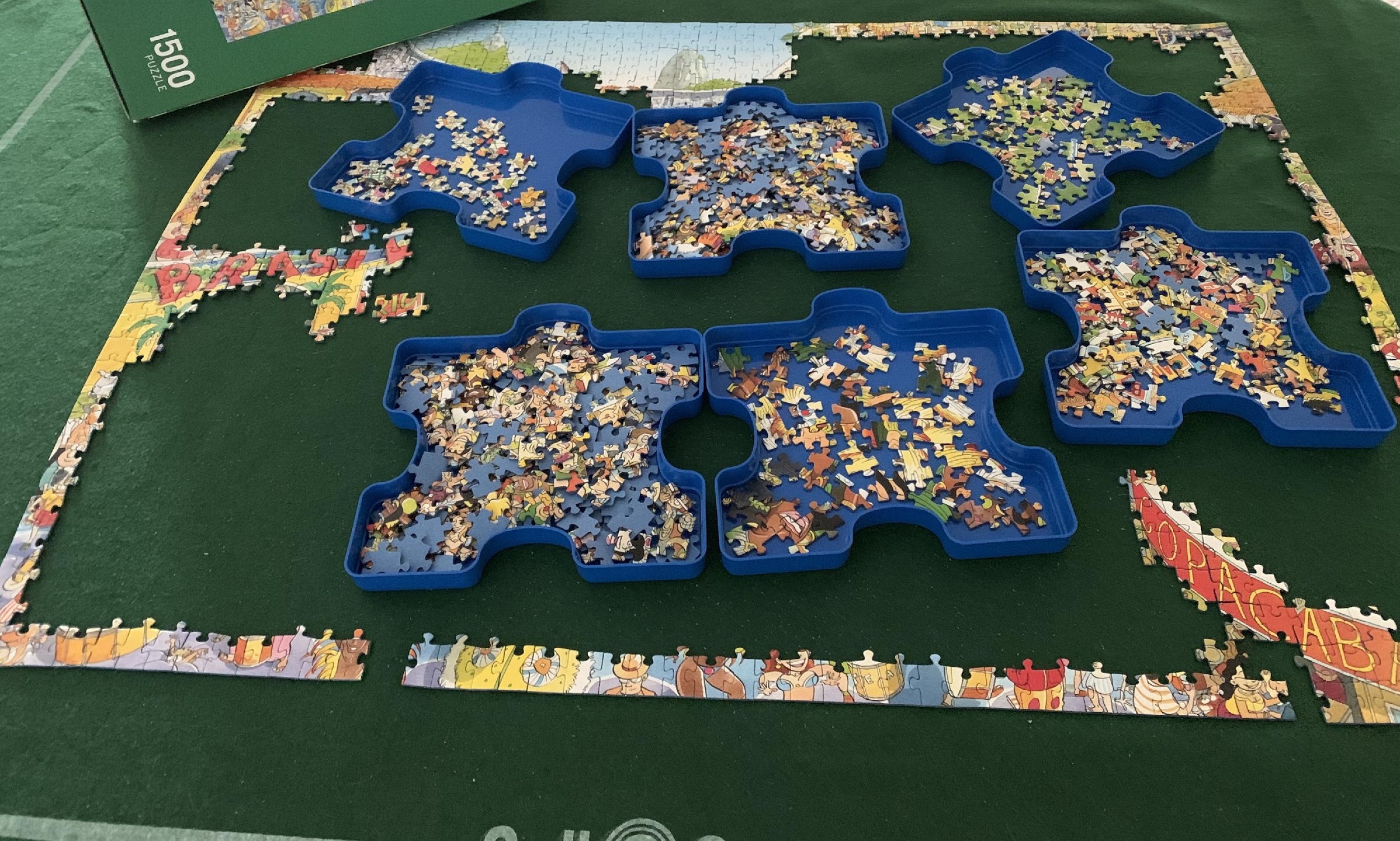** | Numerous puzzle pieces in puzzle sorters surrounded by an incomplete puzzle. | *“Un-puzzling.”* |
| **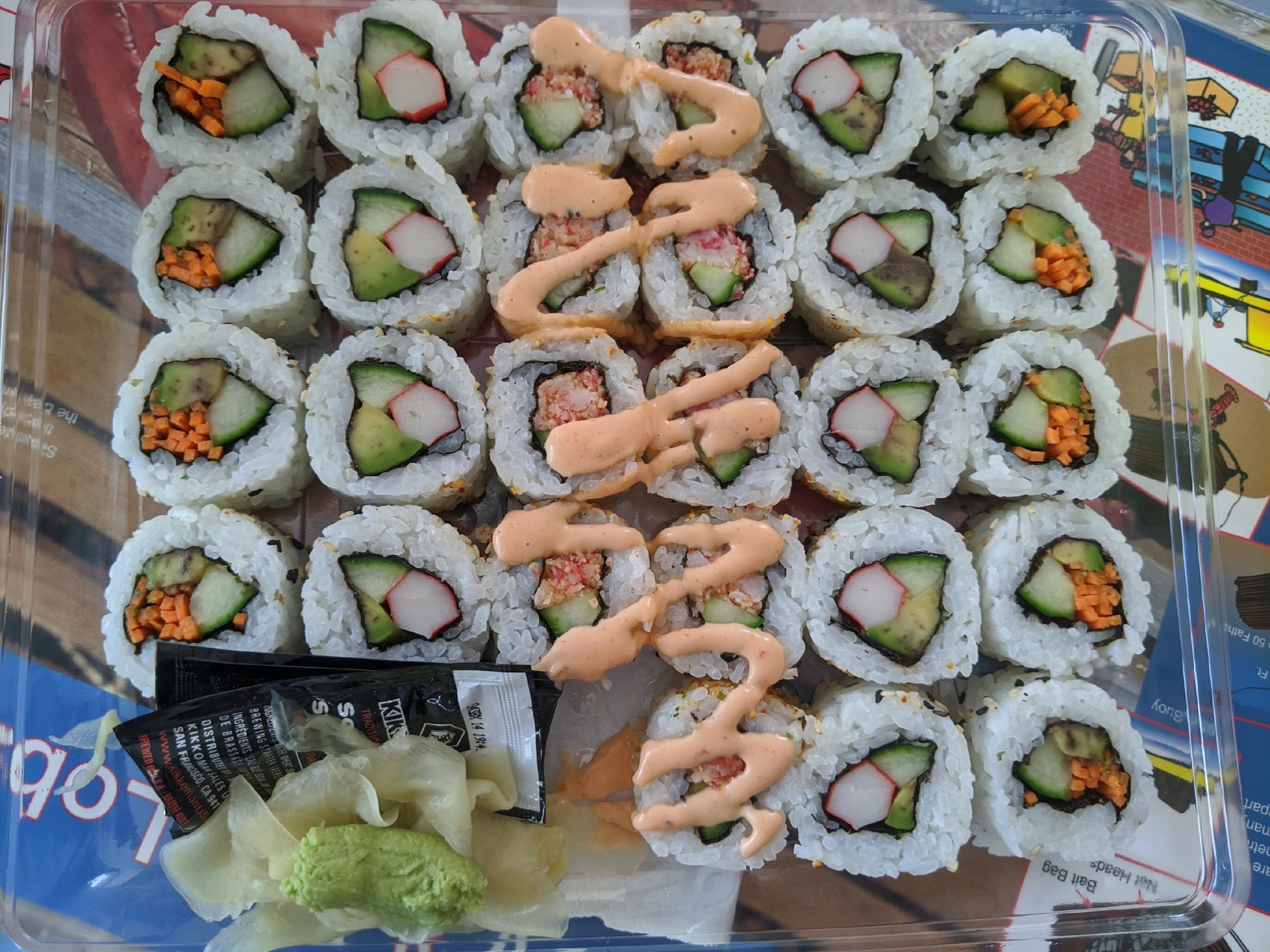** | Packaged sushi with sauce on it. | *“A mother’s love.”* |
| **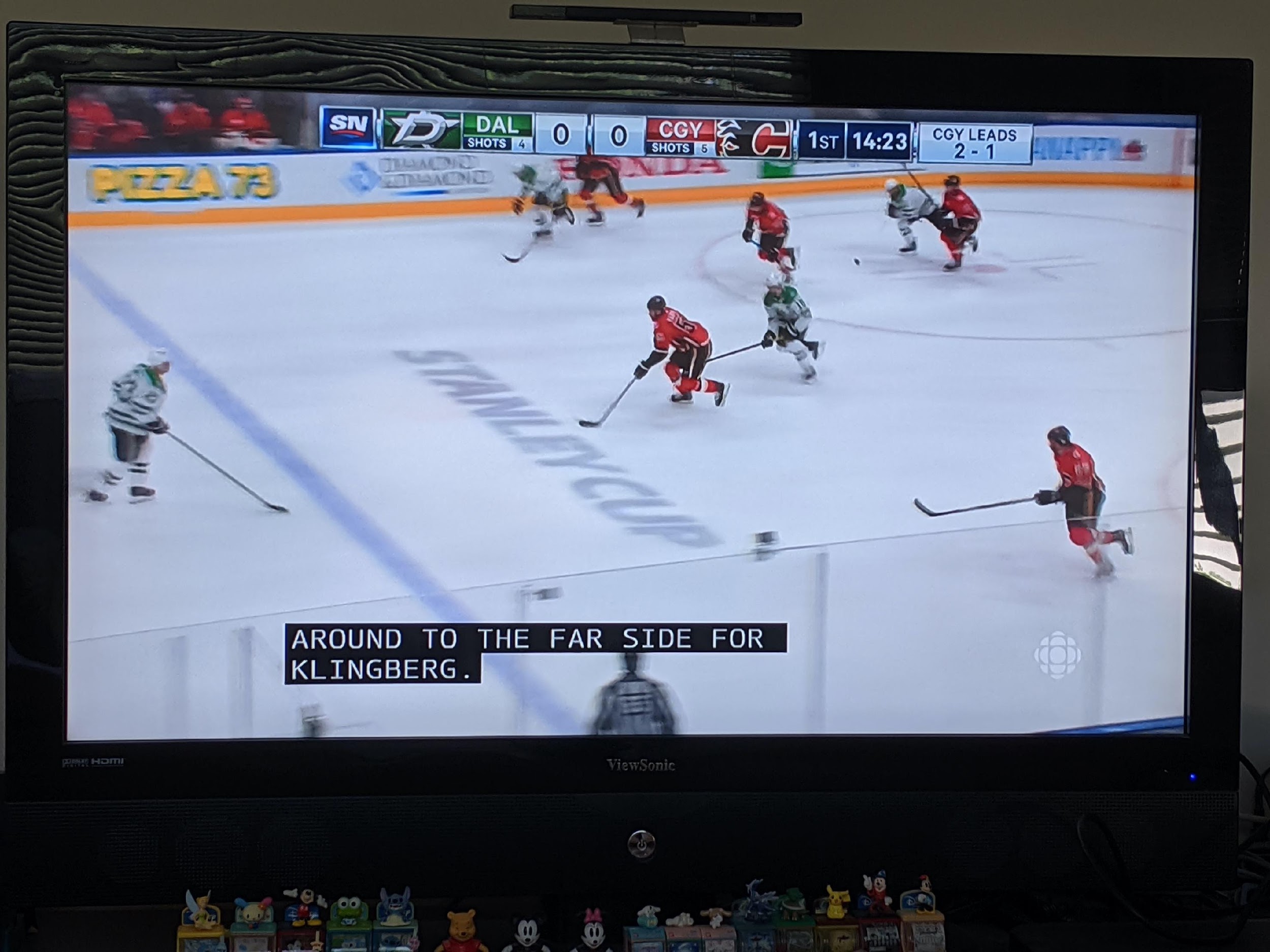** | TV screen capturing the first quarter of a hockey game. | *“A hopeful hockey game.”* |
| **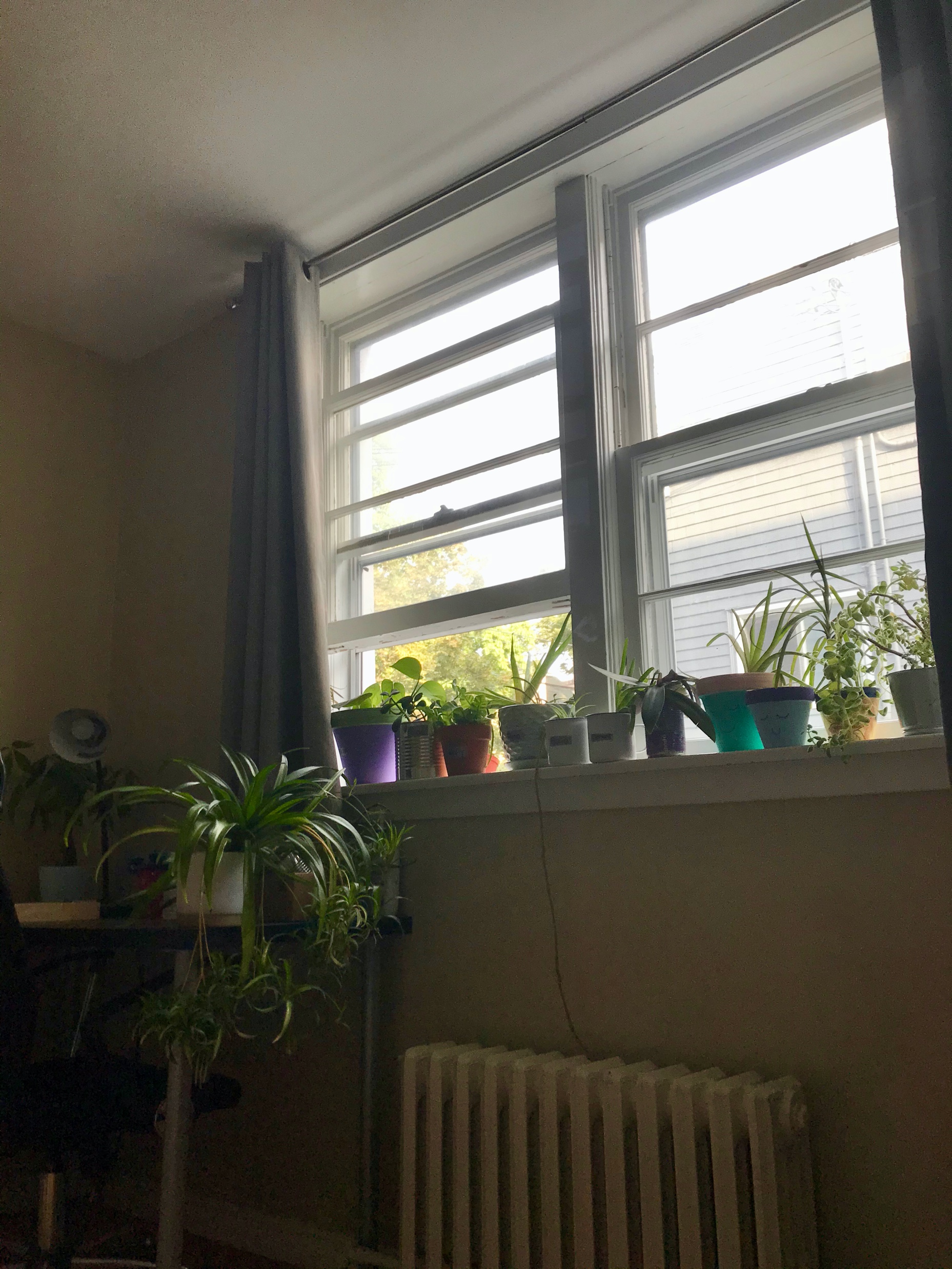** | Numerous plants on a window ledge as the sun shines through. | *“Patience.”* |
| 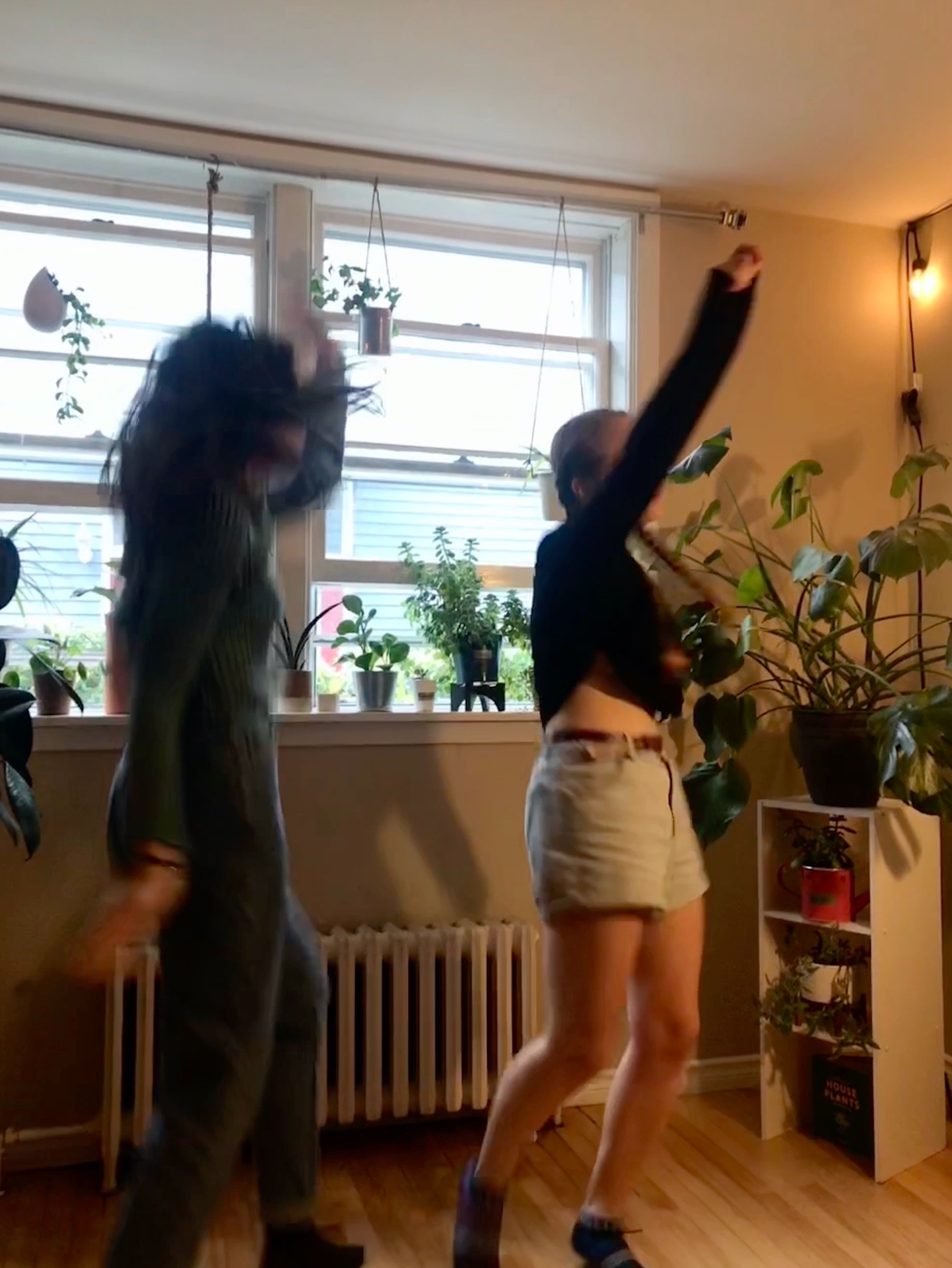 | Two individuals dancing in front of a background of plants sitting a window sill. | *“Two peas in a wonky little pod.”* |
| **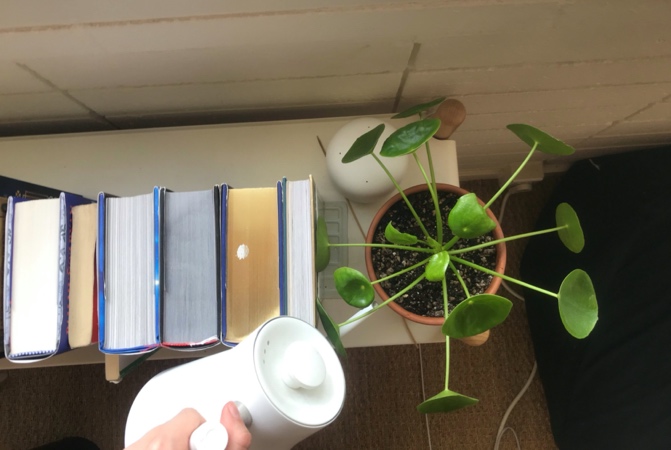** | Water can hovering above a plant, next to a row of books. | *“Taking care of other life.”* |
| Photo removed as participant may be identifiable | Kitchen background with open cabinets with a blurred out individual in the foreground. | *“Not all the way there, but still able to contribute.”* |
| Photo removed as participant may be identifiable | Young woman with her back facing the camera sits on a dock ledge, while looking out to the water. | *“Looking to the future.”* |
| **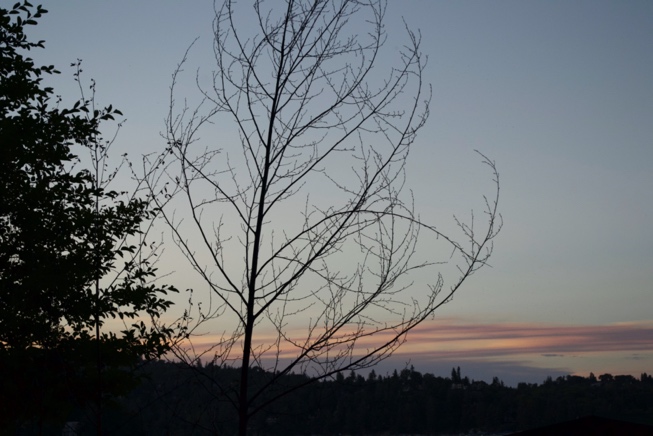** | Silhouette of a tree mostly devoid of any leaves, next to a tree full of leaves as the sun sets in the background. | *“Sunset road.”* |
| **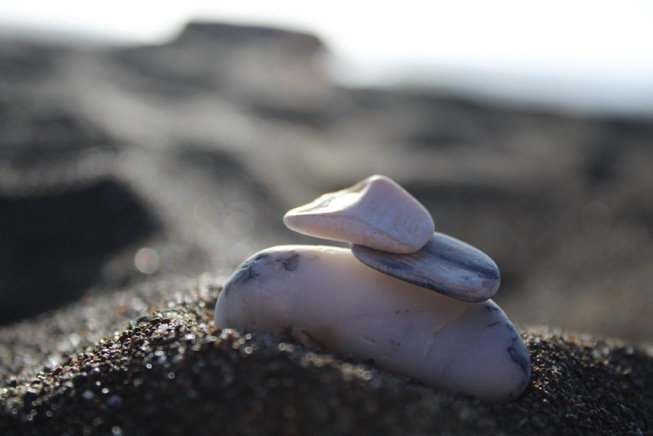** | Three rocks of different sizes balanced upon each other in a pile of dirt. | *“Balancing time.”* |
| **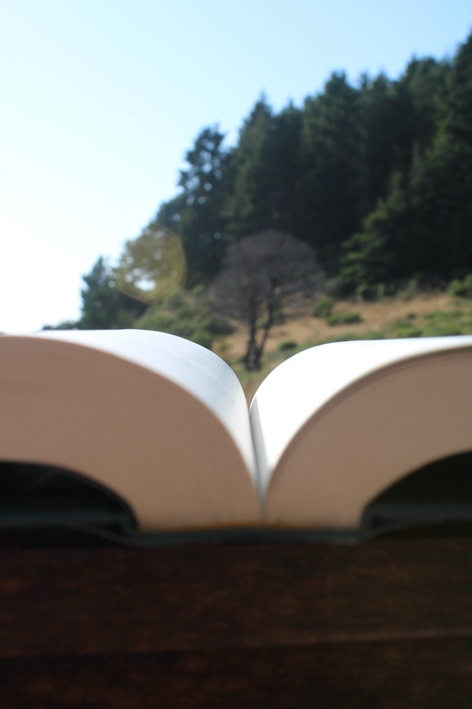** | Open book with trees in the background, with one distinct tree in the center looking as if it’s growing from the book. | *“A world within words.”* |
| **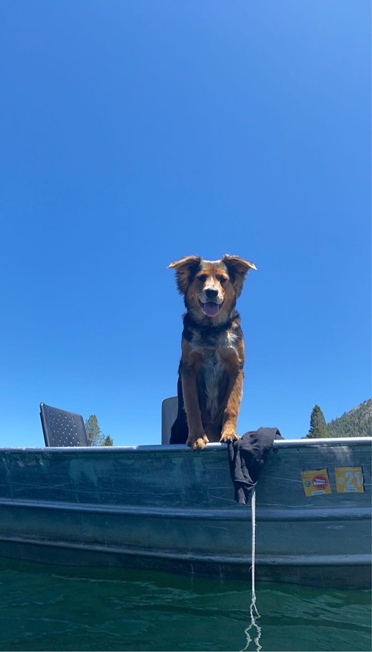** | Dog sitting on a boat ledge that is on the water. | *”Happiness.”* |
| Photo removed as participant may be identifiable | Two individuals tubing on the water with the mountain and partially cloudy sky in the background. | *“Not giving up.”* |
| **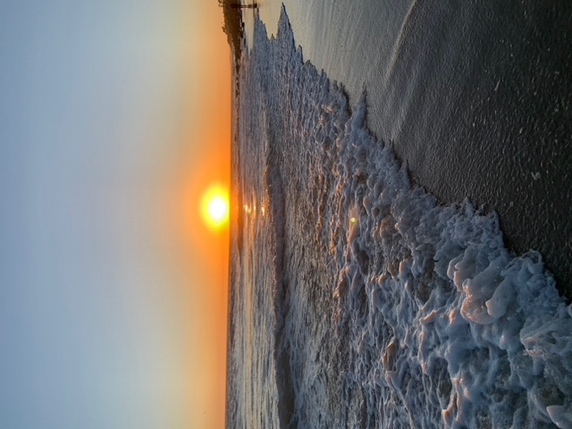** | Sun setting on the beach with the ocean tide coming in. | *“The power of water.”* |
| **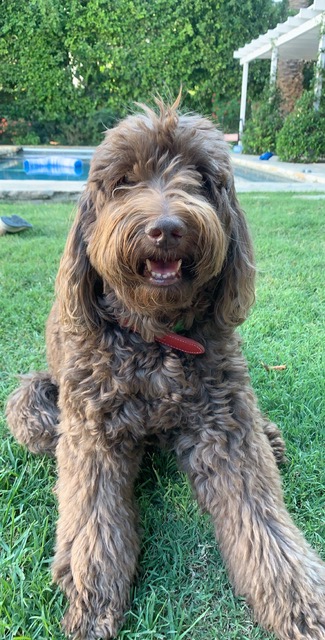** | Brown dog smiling while lying in the grass. | *“The ones around you that can help.”* |
| **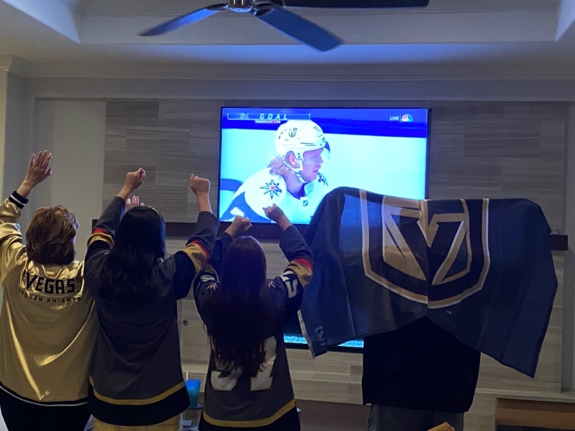** | Four individuals cheering with their hands up in the air with a hockey game playing on the TV. | *“Family bond.”* |
| **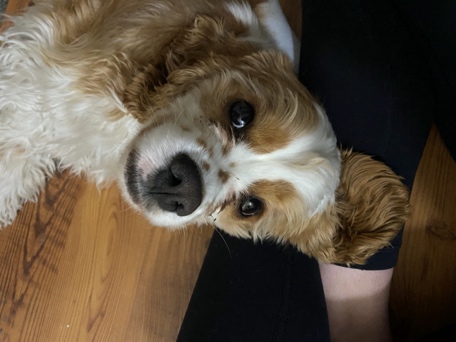** | Brown and white spotted dog staring at the camera. | *“Unconditional love.”* |
| **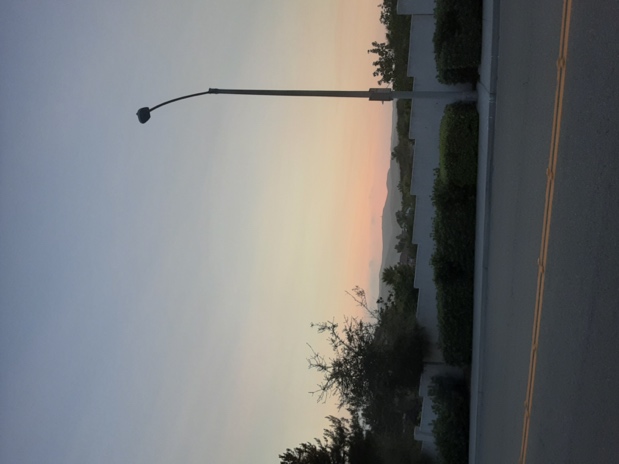** | Empty road with the sun setting in the background with the silhouette of a street lamp in the background. | *“A calming canopy.”* |
| **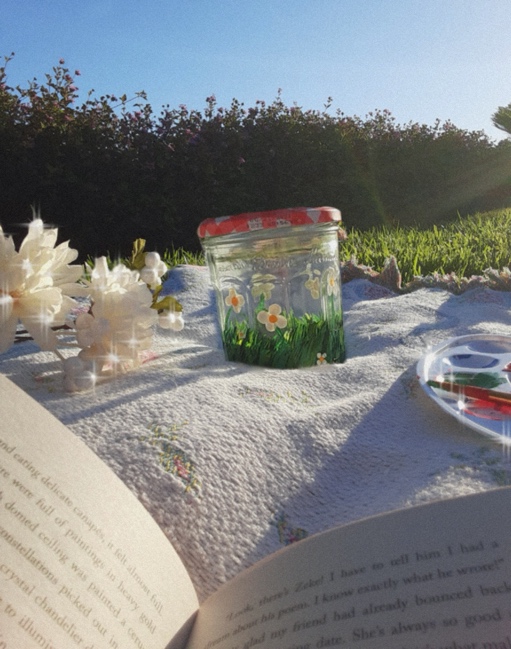** | Open book lying on a blanket with a freshly, colorfully painted mason jar in front with the forest illuminated by the sun in the background. | *“Filled with warmth.”* |
| 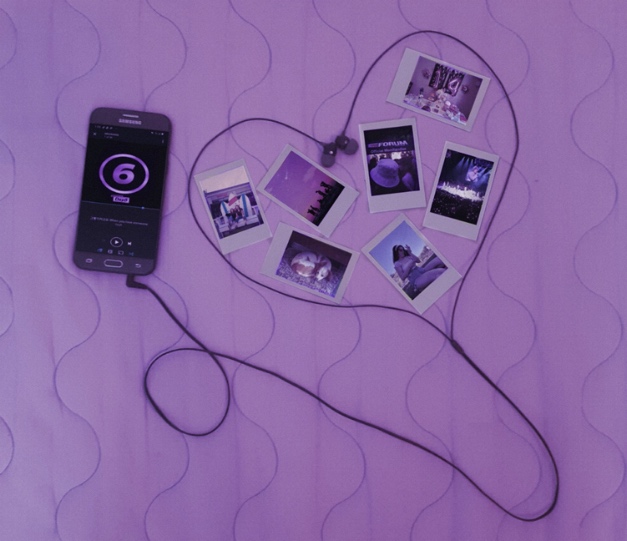 | Cell phone playing music with headphones plugged in, creating the shape of a heart with numerous polaroid photos within the heart. | *“Memory therapy.”* |
| **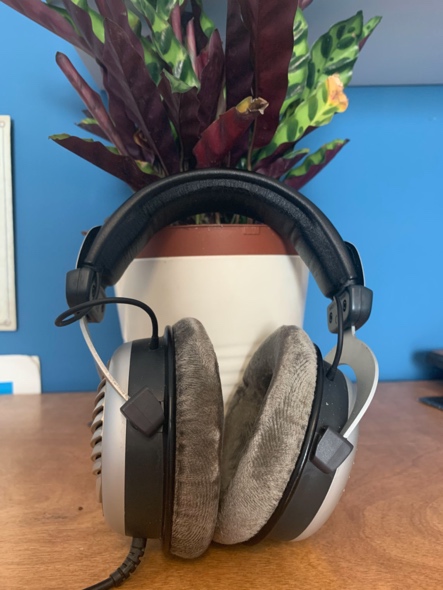** | Over the ear headphones standing up in front of a plotted plant. | *”Empowerment in a simple form.”* |
| **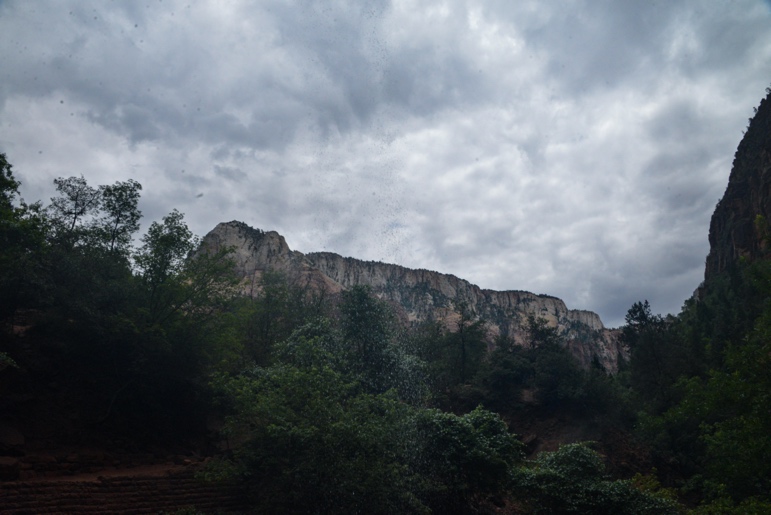** | Large mountain in the forest against the background of a cloudy sky. | *“A Line Between Reality and Fantasy.”* |
