# Supplementary material for: The acceptability of photovoice as a method for incorporating resilience-enhancing factors into pediatric pain research
Source: PLoS One. 2022 Sep 2;17(9):e0272504. doi: 10.1371/journal.pone.0272504 (PMC9439202; doi:10.1371/journal.pone.0272504)
Supplement: S3 File — (PDF) [file pone.0272504.s003.pdf]

Date: 6/12/2020

Study Title: Using Photographs to Share What Helps Young People Living with Chronic Pain

Principal Investigator: Elizabeth Donovan, PhD, Department of Psychology, Simmons University, Boston, MA, and member of the Clinical Advisory Board for Creative Healing for Youth in Pain.

Why are you trying to learn? We want to learn what young people do to help them manage chronic pain. We also want to learn how they feel about taking photos to show what helps them to manage pain. You are being asked to be in the study because you are between 13 and 25 years of age and live with chronic pain.

What will I be asked to do? You will be asked to attend two online group meetings. The meetings will be conducted using free Zoom software. The first meeting will be 90 minutes long. During this meeting, you will be asked to talk in a group about the goal of the study. You will also be given information about taking photos over a two-week period. The second meeting will last for two hours. You will be asked to talk about two of your photos in a group. You will also be asked to talk about photos taken by the other young adults and teens. Finally, you will be asked to talk about what it was like to take the photographs. Elizabeth Donovan, PhD, and Georgia Weston, MSW, will co-lead the groups. Katherina Tanson, a medical student, will take notes. Up to six study participants, and parents of people under 18 years of age, will also be at the meeting. Video of the two meetings will be recorded and later transcribed.

Are there any risks? Photos and words that you share may be displayed in an exhibit or online. The information may also be published or used in grant applications. Your name will never be used. However, you should only submit photographs that you are comfortable being exhibited or published. It is possible that you may be uncomfortable talking in the group. You can refuse to answer any of the questions. A clinician will be at both group meetings. The clinician is trained to help young people experiencing distress. As with all studies, there is a small chance that someone outside of the study might find out you were in the study. To try and make sure this does not happen, names will be removed from all photos and transcripts. All information from the study will be stored on Dr. Donovan's password-protected computer. We will destroy the video recordings within six months of the meetings.

Are there any benefits? You may enjoy being in the study. We also hope that what we learn from the study may help other young people living with pain.

Is there any cost or compensation? You will spend 90 minutes at the first meeting and two hours at the second meeting. You will also spend time taking photos. We will thank you with a $30 Amazon gift card for coming to the first meeting. We will also thank you with a $50 Amazon gift card for coming to the second meeting. Gift cards will be emailed to you at the beginning of each meeting.

How will the photos and the things I say be used? Identifying information will be removed from photos, captions of photos, and transcripts of group discussions. The information may be then used in publications and grant applications. Photos and captions may also be displayed on websites or in exhibits. Information that identifies you will never be displayed with the photos, captions of photos, or transcripts.

How will you protect my identity? We will ask you to rename themselves on Zoom with a fake name. We will also ask you to not repeat what is said in the group discussions to others. We will be using Zoom for Healthcare software for the online meetings. This software includes features to protect privacy. Photos, captions, and transcripts will be kept confidential to the extent permitted by law. Your name will be removed from transcripts. ID numbers will be used in place of names in the transcripts. Names of any friends, family members, etc. who are mentioned during the discussions will also be removed from transcripts. A key linking ID numbers to participants' names and demographic information will be kept on Dr. Donovan's password-protected computer. Katherina Tanson will sign a confidentiality agreement

before transcribing the group conversations. ID numbers will also be used in all written materials and presentations. Only the three members of the research team will know that you participated in the study. All the recordings will be destroyed within six months of the interviews.

Can I stop being in the study? Participation in the research study is voluntary. You may refuse to answer or skip any questions. You may withdraw from the study at any time without penalty.

Contact Information: If you have any questions about the study, please contact the Principal Investigator, Dr. Elizabeth Donovan, at donoe@simmons.edu or 617-521-2604. If you would like to speak with someone who is not directly involved with the study, you may contact Simmons University's Human Protections Administrator (irbprotocols@simmons.edu) or 617-521-2415. The Human Protections Administrator can answer your questions about rights as a human subject or concerns about the research. If you would like to talk with someone about any feelings you have about the study, you may contact Diana Taylor, PhD., clinical psychologist, board certified in California, at 310 475-5875.

Confidentiality: The researchers will do everything they can to make sure your nobody knows you were in the study. However, because you will be in group meetings, the researchers cannot guarantee confidentiality. Please do not repeat what is said in the group discussions to others.

____I agree to maintain the confidentiality of the information discussed by all participants and researchers during the group meetings.

____I do not agree to maintain the confidentiality of the information discussed by all participants and researchers during the group meetings.

Consent Statement: I have read this consent form. I have had an opportunity to talk about the consent form with the investigator. All my questions have been answered. I understand the goal of the project. I understand what I will be asked to do. I understand the possible risks and benefits of being in the study. I understand that my participation is voluntary. I know that I can stop being in the study at any time without penalty.

I voluntarily give consent to participate in this research study. I have saved or printed a copy of this consent form.

Signature ____

I __________________________________ agree to allow you to video record the two online meetings.

I __________________________________ do not agree to you to video record the two online meetings.

Signatures of investigator ____

Date: 6/12/2020

Study Title: Using Photographs to Share What Helps Young People Living with Chronic Pain

Principal Investigator: Elizabeth Donovan, PhD, Department of Psychology, Simmons University, Boston, MA, and member of the Clinical Advisory Board for Creative Healing for Youth in Pain.

What is the study about? We are interested to learn what helps you to feel better when you have pain. We also want to know how you feel about taking photos of things that help you feel better. You have been invited to be part of the study because you are between 13 and 25 years of age and live with chronic pain.

What will I be asked to do? You will be asked to attend two online group meetings. The first meeting will be 90 minutes long. You will be asked to discuss taking photographs of things that help you feel better in a group. You will then be asked to take photographs over a two-week period. At the second, two-hour meeting, you will be asked to discuss two of your photographs in a group. You will also be asked to talk about photographs taken by the other young people. Lastly, you will be asked to tell us how you felt about taking the photographs. Video of the two meetings will be recorded and later transcribed.

Who will be there? The people present at the meetings will be Elizabeth Donovan, PhD, and Georgia Weston, MSW, who will co-lead the groups; Katherina Tanson, a medical student, who will take notes; up to six participants, and parents of participants under 18 years of age.

Will anything negative happen to me? It is possible that you may be uncomfortable talking about your photos, other people's photos, or what it was like to take the photos. You do not have to answer any of the questions. A clinician will be at the meeting. She will help you if you are uncomfortable. We will remove names from all photos and transcripts and store them on a password-protected compute. We will also destroy video recordings within six months of the meetings.

Will anything positive happen to me? Some people enjoy taking the photos and talking about them. We also hope that what you tell us may help other young people living with pain.

Cost and Compensation. You will spend 90 minutes at the first meeting and two hours at the second meeting. You will also spend time taking photos. We will thank you with a $30 Amazon gift card for coming to the first meeting. We will thank you with a $50 Amazon gift card for coming to the second meeting. Gift cards will be emailed to your parent at the beginning of each meeting.

How will what you share be used in the future? We will remove your name from captions of photos and transcripts of group discussions. We may then write about and publish what you have shared. Photos and captions may also be displayed in exhibits or online. Your name, or any other information that identifies you, will never appear with the photographs, captions of photographs, or transcripts of discussions.

How you will prevent people knowing that I was in the study? We will do everything we can to make sure that only the three members of the research team know that you were part of the study. We will ask you to rename yourself on Zoom with a fake name. We will also ask you not repeat what is said in the group to others. We will use video software that has features to protect privacy. Your name will be removed from transcripts of discussions and the photos you shared. All information will then be stored on a password-protected computer. All the recordings will be destroyed within six months of the interviews.

Do I have to participate? No. You do not have to participate. You can refuse to answer or skip any questions. You can also stop being part of the study at any time. This will not affect anything.

Who can I talk to about the study? If you have any questions about the research, please contact the Principal Investigator, Dr. Elizabeth Donovan, at donoe@simmons.edu or 617-521-2604. If you would like to speak with someone who is not directly involved with the study, you may contact Simmons University's Human Protections Administrator for questions about rights as a human subject or concerns about the research (irbprotocols@simmons.edu) or 617-521-2415. If you would like to talk with someone about any feelings you have about being in the study, you may contact Diana Taylor, PhD., clinical psychologist, board certified in California, at 310 475-5875.

Confidentiality: The researchers will do everything they can to make sure your nobody knows you were in the study. However, because you will be in group meetings, the researchers cannot guarantee confidentiality. Please do not repeat what is said in the group discussions to others.

____I agree to maintain the confidentiality of the information discussed by all participants and researchers during the group meetings.

____I do not agree to maintain the confidentiality of the information discussed by all participants and researchers during the group meetings.

Volunteering to be in the study: I have read this form and have had an opportunity to have my questions answered. I understand what will happen in the study and the plusses and minuses to being part of the study. I understand that I do not have to be part of the study. I can change my mind any time and nothing bad will happen.

I am volunteering to participate in this research study. I have saved or printed a copy of this form.

Signature

____

I ____ agree to allow you to video the two online meetings.

I ____ do not agree to you to video the two online meetings.

Signatures of investigator

____

Date: 6/12/2020

Study Title: Using Photographs to Share What Helps Young People Living with Chronic Pain

Principal Investigator: Elizabeth Donovan, PhD, Department of Psychology, Simmons University, Boston, MA, and member of the Clinical Advisory Board for Creative Healing for Youth in Pain.

Why are you trying to learn? We want to learn what young people do to help them manage chronic pain. We also want to learn how they feel about taking photos to show what helps them to manage pain. Your teen is being asked to be in the study because they are between 13 and 25 years of age and live with chronic pain.

What will my teen be asked to do? You and your teen will be asked to attend two online group meetings. The meetings will be conducted using free Zoom software. We would like you to be with your teen for both meetings. The first meeting will be 90 minutes long. During this meeting, your teen will be asked to talk in a group about the goal of the study. Your teen will also be given information about taking photos over a two-week period. The second meeting will last for two hours. Your teen will be asked to talk about two of their photos in a group. They will also be asked to talk about photos taken by the other teens and young adults. Finally, your teen will be asked to talk about what it was like to take the photographs. Elizabeth Donovan, PhD, and Georgia Weston, MSW, will co-lead the groups. Katherina Tanson, a medical student, will take notes. Up to six study participants, and parents of people under 18 years of age, will also be at the meeting. Video of the two meetings will be recorded and later transcribed.

Are there any risks? Photos and words shared by your teen may be displayed in an exhibit or online. The information may also be published or used in grant applications. Your teen's name will never be used. However, your teen should only submit photographs that they are comfortable being exhibited or published. It is possible that your teen may be uncomfortable talking in the group. Your teen can refuse to answer any of the questions. A clinician will be at both group meetings. The clinician is trained to help young people experiencing distress. As with all studies, there is a small chance that someone outside of the study might find out your teen was in the study. To try and make sure this does not happen, names will be removed from all photos and transcripts. All information from the study will be stored on Dr. Donovan's password-protected computer. We will destroy the video recordings within six months of the meetings.

Are there any benefits? Your teen may enjoy being in the study. We also hope that what we learn from the study may help other young people living with pain.

Is there any cost or compensation? Your teen will spend 90 minutes at the first meeting and two hours at the second meeting. Your teen will also spend time taking photos. We will thank your teen with a $30 Amazon gift card for coming to the first meeting. We will also thank your teen with a $50 Amazon gift card for coming to the second meeting. Gift cards will be emailed to you at the beginning of each meeting.

How will the photos and the things my teen says be used? Identifying information will be removed from photos, captions of photos, and transcripts of group discussions. The information may be then used in publications and grant applications. Photos and captions may also be displayed on websites or in exhibits. Information that identifies your teen will never be displayed with the photos, captions of photos, or transcripts.

How will you protect the identity of my teen? We will ask your teen to rename themselves on Zoom with a fake name. We will also ask them to not repeat what is said in the group discussions to others. We will be using Zoom for Healthcare software for the online meetings. This software includes features to

protect privacy. Photos, captions, and transcripts will be kept confidential to the extent permitted by law. Your teen's name will be removed from transcripts. ID numbers will be used in place of names in the transcripts. Names of any friends, family members, etc. who are mentioned during the discussions will also be removed from transcripts. A key linking ID numbers to participants' names and demographic information will be kept on Dr. Donovan's password-protected computer. Katherina Tanson will sign a confidentiality agreement before transcribing the group conversations. ID numbers will also be used in all written materials and presentations. Only the three members of the research team will know that your teen participated in the study. All the recordings will be destroyed within six months of the interviews.

Can my teen stop being in the study? Participation in the research study is voluntary. Your teen may refuse to answer or skip any questions. Your teen may withdraw from the study at any time without penalty.

Contact Information: If you have any questions about the study, please contact the Principal Investigator, Dr. Elizabeth Donovan, at donoe@simmons.edu or 617-521-2604. If you would like to speak with someone who is not directly involved with the study, you may contact Simmons University's Human Protections Administrator (irbprotocols@simmons.edu) or 617-521-2415. The Human Protections Administrator can answer your questions about rights as a human subject or concerns about the research. If you would like to talk with someone about any feelings you have about the study, you may contact Diana Taylor, PhD., clinical psychologist, board certified in California, at 310 475-5875.

Confidentiality: The researchers will do everything they can to make sure your nobody knows your teen was in the study. However, because your teen will be in group meetings, the researchers cannot guarantee confidentiality. Please do not repeat what is said in the group discussions to others.

____I agree to maintain the confidentiality of the information discussed by all participants and researchers during the group meetings.

____I do not agree to maintain the confidentiality of the information discussed by all participants and researchers during the group meetings.

Consent Statement: I have read this consent form. I have had an opportunity to talk about the consent form with the investigator. All my questions have been answered. I understand the goal of the project. I understand what my teen will be asked to do. I understand the possible risks and benefits of being in the study. I understand that my teen's participation is voluntary. I know that my teen can stop being in the study at any time without penalty.

I voluntarily give consent for my teen to participate in this research study. I have saved or printed a copy of this consent form.

Signature ____

I __________________________________ agree to allow you to video record the two online meetings.

I __________________________________ do not agree to you to video record the two online meetings.

Signatures of investigator ____
